# A Pilot Study on the Prevalence and Characterization of Multidrug‐Resistant Gram‐Negative Bacteria in Chicken and Pork Meat Around Kathmandu District, Nepal

**DOI:** 10.1002/mbo3.70275

**Published:** 2026-03-27

**Authors:** Sushma Paudel, Sumitra Tamang, Preety Singh, Rishav Das, Sushmita Paudel, Astha Shakya

**Affiliations:** ^1^ Himalayan WhiteHouse International College Kathmandu Bagmati Nepal; ^2^ Wright State University Dayton Ohio USA

**Keywords:** antimicrobial resistance (AMR), antimicrobial susceptibility test (AST), clinically significant antibiotics, food‐borne pathogen, multidrug resistance (MDR)

## Abstract

The increasing trend of multidrug resistance in Gram‐negative bacteria (MDR‐GNB) has been a major concern for the healthcare settings in Nepal and globally. In Nepal, previous studies on antimicrobial resistance (AMR) have received limited attention to animal‐sourced food, which represents a significant route for the zoonotic transmission of antibiotic‐resistant pathogens. This study aimed to assess antibiotic resistance and multidrug resistance patterns of Gram‐negative bacteria isolated from intestinal and gizzard samples of chicken and pork meat collected from five municipalities in Kathmandu. From 13 samples (11 chicken and 2 pork), a robust collection of 136 Gram‐negative bacteria was isolated and subjected to susceptibility testing against 16 different antibiotics. The bacterial isolates, representing 11 distinct types, were successfully identified: *Escherichia coli* (33.82%) and *Shigella* spp. (11.76%) were the predominant bacteria, followed by *Yersinia* (8.8%), *Citrobacter* (8.8%), *Proteus* (8.8%), *Klebsiella* (5.1%), and *Plesiomonas* (5.1%), among others. Notably, the prevalence of multidrug‐resistant bacteria was notably high, with half (50%) of the isolates exhibiting resistance to multiple classes of antibiotics. Additionally, 73.9% of *E. coli*, 14.28% of *Klebsiella*, 58.33% of *Yersinia*, and 41.67% of *Citrobacter* are found to be multidrug resistant. Thus, this study showed that the meat samples exhibited a high burden of MDR‐GNB, indicating a high risk of food‐borne illness and zoonotic transmission, calling for AMR monitoring and mitigation strategies.

## Graphical abstract

This study investigated multidrug resistance in Gram‐negative bacteria from chicken and pork meat in Kathmandu, isolating 136 strains from 13 samples. Nearly half were multidrug resistant, highlighting contaminated meat as a potential route for zoonotic transmission and the need for antimicrobial resistance monitoring in Nepal.

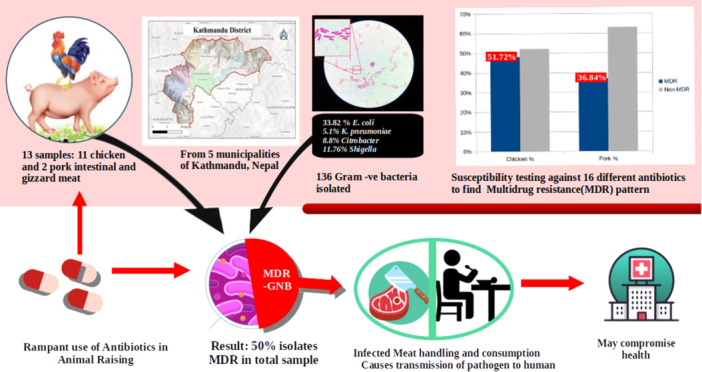

**Abbreviations**
AMR: antimicrobial resistance
AST: antimicrobial susceptibility test
ATCC: American Type Culture Collection
CLSI: Clinical and Laboratory Standards Institute
MAR: multiple antimicrobial resistance
MDR: multidrug resistance
MDRGNs: multidrug‐resistant Gram‐negative organisms
°C: degree celsius
μg: microgram

## 1 Introduction

### 1.1 Background and Significance of the Problem

According to the WHO, antibiotics are medicines used to prevent and treat bacterial infections. Antibiotics, a revolutionary medical discovery, play a crucial role in saving millions of lives by treating and preventing the spread of various bacterial infections (K. P. Acharya and Wilson 2019). Based on the mechanism of antimicrobial activity, antimicrobial agents are classified into groups as cell wall synthesis inhibitors, agents that depolarize the cell membrane, protein synthesis inhibitors, nucleic acid synthesis inhibitors, and metabolic pathways inhibitors in bacteria (Reygaert 2018). There are classes of antibiotics like Beta‐lactams, Tetracyclines, Chloramphenicol, Quinolones and Fluoroquinolones, Carbapenems, Cephalosporins, Macrolides, Sulfonamides, and so forth (Kapoor et al. 2017). Despite the diverse mechanisms of action of these agents, suboptimal antimicrobial stewardship has facilitated the emergence of significant resistance (K. P. Acharya and Wilson 2019).

Antimicrobial resistance (AMR) has emerged as a pressing worldwide health concern, killing at least 1.27 million people worldwide and associated with nearly 5 million deaths in 2019, according to a report released by Center for Disease Control and Prevention (CDC) (Murray et al. 2022). In the same year, six primary pathogens, namely *Escherichia coli*, *Staphylococcus aureus*, *Klebsiella pneumoniae*, *Streptococcus pneumoniae*, *Acinetobacter baumannii*, and *Pseudomonas aeruginosa*, were reported to have caused roughly 74% of AMR‐associated deaths (Murray et al. 2022). The available research evidence shows that resistance of some Gram‐negative organisms against commonly used antibiotics is continuously increasing (Tamma et al. 2012; Fair and Tor 2014; Giske et al. 2008; Centers for Disease Control and Prevention 2011). It is particularly concerning because these Gram‐negative bacteria have a high potential to find new ways to be resistant and can pass along genetic materials to other bacteria via horizontal gene transfer that can make them antibiotic‐resistant (Centers for Disease Control and Prevention 2011). Additionally, the emerging trend of multidrug‐resistant Gram‐negative organisms (MDRGNs) is the major threat to the infected and hospitalized patients, which has raised the mortality rate to 30%–70% (Tamma et al. 2012; Fair and Tor 2014). Notably, *E. coli*, a Gram‐negative bacterium, was the leading cause of AMR‐attributable deaths, followed by *K. pneumoniae* and *S. aureus* (Murray et al. 2022). This threat is compromising the efficacy of antibiotics and presenting substantial hazards to both the healthcare system and patient well‐being (Centers for Disease Control and Prevention 2011).

Parallel trends are evident in Nepal, where the burden of AMR is exacerbated by the unregulated use of antibiotics and inadequate infection control measures. Several studies (Madhup et al. 2021; Saud et al. 2019; Koju et al. 2022) have shown an increase in resistance to common antibiotics in different bacterial species.

Meat (the food of animal origin) is rich source of nutrition like amino acids, peptides and proteins (nitrogenous compound), minerals and growth factors and is high in moisture content which makes it the ideal medium for the growth of numerous microorganisms which makes them the predominant source for human acquired *Campylobacter* species and nontyphoidal *Salmonella*, *Enterococcus*, *Pseudomonas*, and other commensal bacteria causing infection (Thanigaivel and Swedha Anandhan 2015; Collignon et al. 2016). To exacerbate the situation, there is an increasing trend of meat consumption in Nepal that showed the rise of meat consumption from 375,000 tons in 2019 to 552,000 tons in 2020 per year (Nepal 2022), and on average, each Nepalese person consumes 18 kg of meat annually. The data also shows that poultry meat accounts for 46% of the total meat consumption in Nepal, followed by buffalo meat at 33%, goat meat at 17%, and pork at 4% (Nepal 2022). With the increasing trend of meat consumption, there is a need for the production of meat products to meet the market demand. To fulfill this huge demand, the indiscriminate use of antibiotics for prophylactic purposes is observed (Madhup et al. 2021). Moreover, in commercial animal farming, overcrowding often leads to an increased risk of disease transmission. To counteract this, antibiotics are frequently administered for therapeutic purposes (Madhup et al. 2021). This has contributed to the emergence and spread of antibiotic‐resistant bacteria like *E. coli* and *Salmonella* (WHO 2021). Most importantly, resistance genes among bacteria in food animals may also spread to humans via contaminated food, water, or direct contact, and further spread of these genes to bacteria carried by humans is particularly a serious threat (Collignon et al. 2016; K. P. Acharya and Wilson 2019).

In the context of Nepal, multiple antibiotic‐resistant Enterobacteriaceae from meat samples, litter in animal farms, and sludges of slaughterhouses have also been studied and documented (Madhup et al. 2021; Saud et al. 2019; Shrestha et al. 2017). In 2022, researchers found high rates of AMR in Gram‐negative *E. coli* isolated from chicken meat samples collected from local markets in Dhulikhel Municipality, Nepal, with more than 86% of the *E. coli* isolates being resistant to tetracycline and 66% resistant to ciprofloxacin (Koju et al. 2022). There has been an increasing trend of the MDR‐GNB in meat products. Nearly 50% of the isolates are observed to be MDR in the study of Magiorakos et al. (2012).

The rise of resistance emphasizes the need to understand its mechanisms and to research new therapeutic avenues (Kapoor et al. 2017). With these concerning trends, our research endeavors center on understanding the landscape of multidrug‐resistant Gram‐negative bacteria.

## 2 Research Design and Methodology

### 2.1 Sampling and Sample Preparation

**Figure 1:**
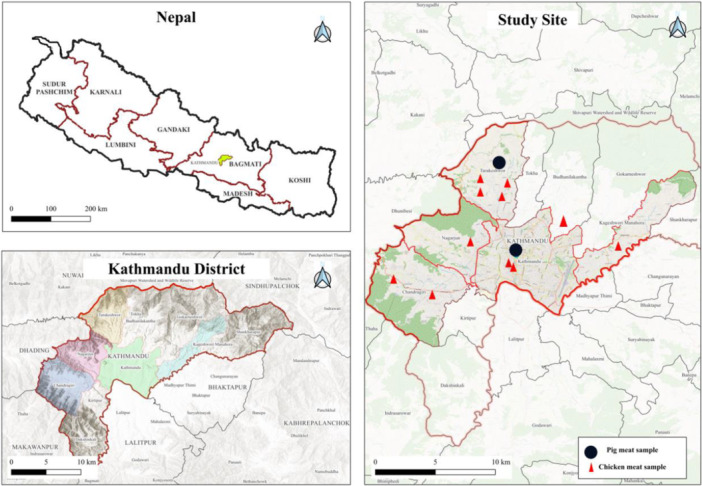
Sampling site within Kathmandu district of Nepal.

**Figure 2:**
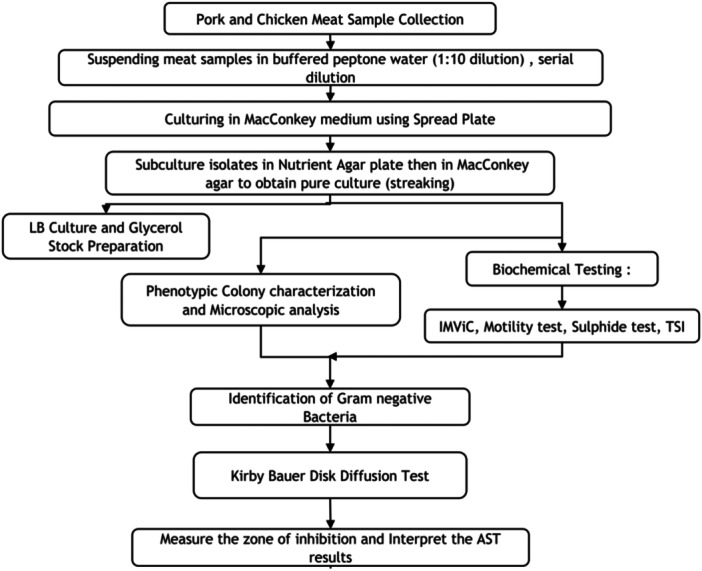
Flowchart of methodology.

In this cross‐sectional pilot study, a total of 11 chicken and 2 pork intestinal and gizzard meat samples from meat shops and slaughterhouses in Kathmandu city (schematic sampling site: Figure 1) were collected between April and June 2023 for microbial isolation and identification. Due to the exploratory nature of this research, a convenience sampling method was employed to provide a snapshot of AMR in retail environments. While this method may introduce selection bias, it serves as a critical baseline for future randomized surveillance. Intestinal and gizzard tissues were processed independently for each sample to facilitate comparative microbial isolation. The processes involved are shown in Figure 2.

Samples were collected in a sterile plastic bag, sealed, and labeled with meat sample identifiers (organism name, shop name and location, and date and time of collection). 25–50 g of samples were obtained by taking the whole intestine and gizzard of chicken, while for pork, 150–200 g samples were taken. Samples were kept in the ice box immediately upon collection and transported to the laboratory of Himalayan WhiteHouse International College within 2 h and stored at 0°C–4°C for no longer than 36 h, until the isolation was carried out (Andrews and Hammack 2003; FDA 2022).

Samples were prepared for microbiological determination, strictly adhering to aseptic procedures as described in Benson's Microbiological Application and Laboratory Manual for both plate and broth culturing (Brown and Smith 2005, 65–84). The intestine and gizzard of the meat sample were aseptically chopped, separated, cut into small pieces, and weighed. A 15 g aliquot of each sample was homogenized in 135 mL of sterile buffered peptone water (pH 7.0) to achieve a 1:10 dilution. Since the intestinal samples were, in most cases, less than 25 g in total, the 15 g sample was used for further analysis. The rinsate from the prepared sample was used to form the test suspension. Subsequent serial dilution was carried out to create the desired dilution level of up to 10^−6^.

### 2.2 Microbial Isolation and Characterization

#### 2.2.1 Spread Plate Culture in MacConkey Agar Medium and Subculture

**Figure 3:**
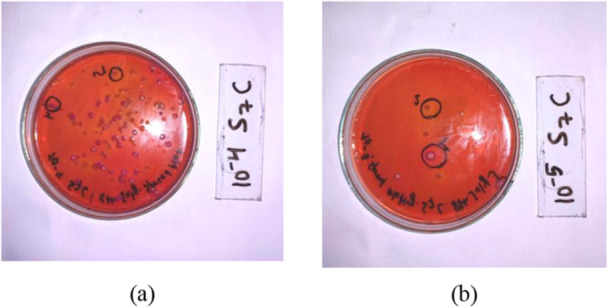
Isolation of colonies by spread plating in MacConkey and colonies with different morphological characteristics. (a) isolation in 10^‐4 dilution, (b) isolation in 10^‐5 dilution.

For each of the sample's dilutions (10^−3^ to 10^−6^), spread plating was performed in sterile dried plates of MacConkey agar using a volume of not less than 0.1 mL of the sample rinsate (Allen 2005; Wise 2006). The plates were incubated at 37°C for 24 h to obtain the isolated colonies of Gram‐negative bacteria as shown in Figure 3.

**Figure 4:**
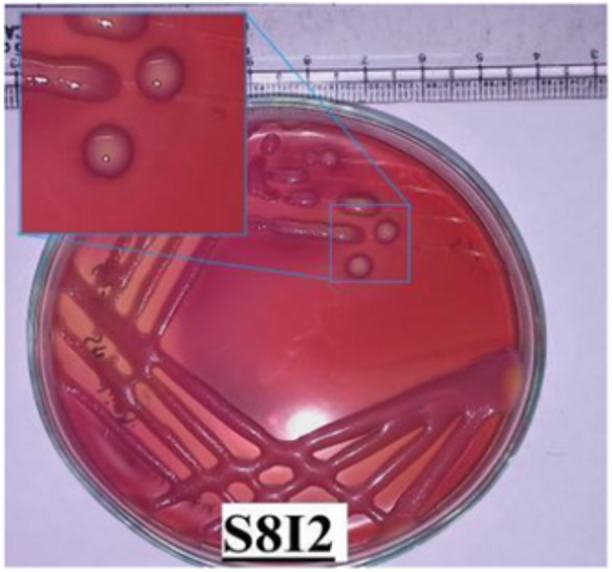
Colony morphology of chicken meat sample: Pink, circular, entire, convex, mucoid, opaque colony of *Klebsiella* spp.

From plates displaying 20–200 colonies, 10–12 distinct colonies were selected from each meat sample based on morphological characteristics and relative abundance. These were then subcultured on sterile Nutrient Agar (NA) plates using the streaking technique as in Figure 4. After the initial streaking on NA plates, the colonies were carefully transferred to MacConkey Agar plates, followed by rigorous morphological assessment of the pure culture isolates obtained after incubation for 24 h (Allen 2005; Leboffe and Pierce 2015).

**Figure 5:**
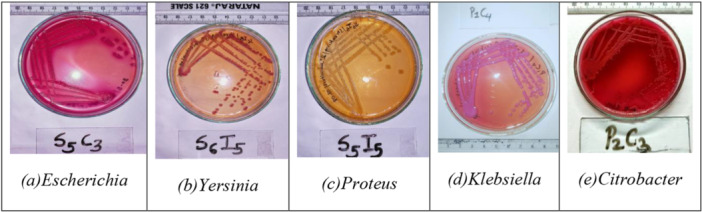
Colony morphology in MacConkey agar.

Isolates were phenotypically characterized based on colony morphology, including size, shape, pigmentation on the growth medium, and texture, as shown in Figure 5. These criteria were essential for detailed phenotypic profiling, aiding the precise identification and classification of bacterial strains (Breakwell et al. 2007).

#### 2.2.2 Microscopic Analysis

**Figure 6:**
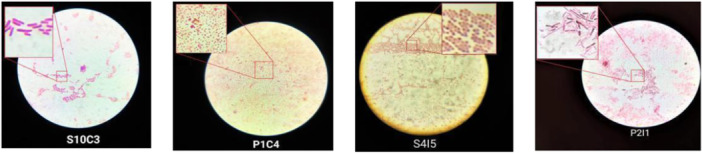
Gram‐staining showing all isolates with pink colored cells under microscope: S10C3 = *Citrobacter* spp., long rod; P1C4 = *Klebsiella* spp., short rod; S4I5 = *Yersinia* spp., bacillus‐cocci; P2I1 = *Escherichia* spp., long rod.

Gram's Iodine test was performed for the morphological characterization of isolated bacterial cells. A single colony was used for the test. The results of the staining procedure were observed under oil immersion using a Brightfield microscope. All the Gram‐negative bacterial cells with the pink stain on Gram's staining were analyzed and reported for their shape, size, and arrangement (some examples are shown in Figure 6).

### 2.3 Biochemical Test

**Figure 7:**
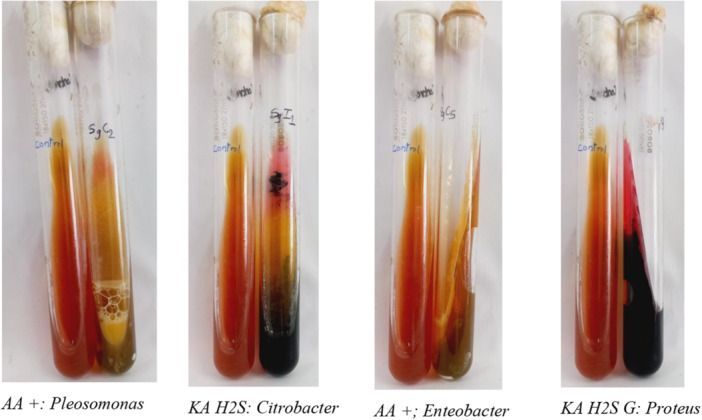
TSI test for glucose, sucrose, lactose fermentation, gas, and sulfide production.

**Figure 8:**
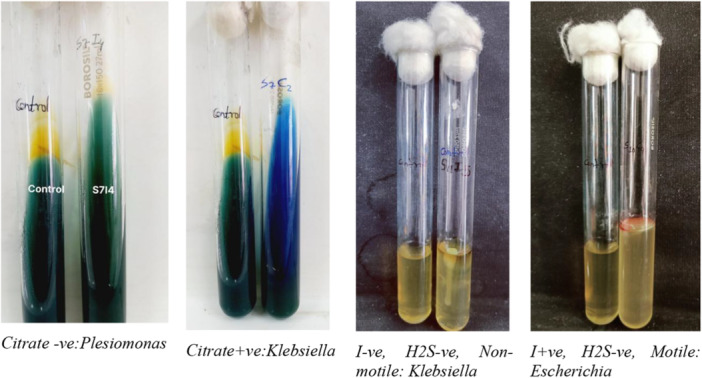
Citrate utilization and SIM test for sulfide, indole, and motility.

**Figure 9:**
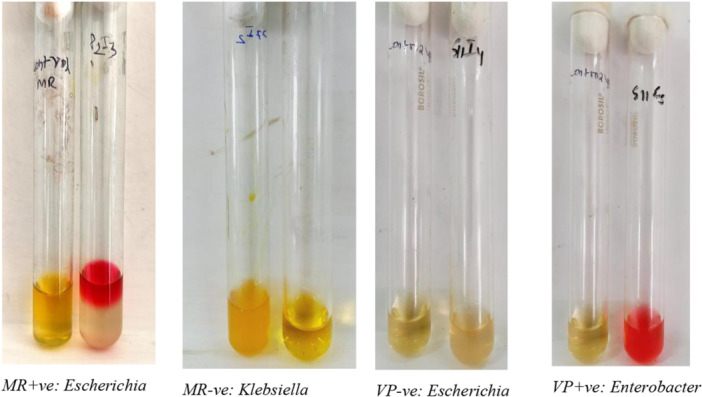
MR‐VP test.

Bergey's Manual of Determinative Bacteriology was used as a guideline for Gram‐negative bacterial identification based on properties like motility, indole production, hydrogen sulfide production, citrate utilization, glucose/sucrose/lactose sugar utilization, gas production, acid production by mixed acid fermentation pathway, and ability to produce 2,3‐butanediol from glucose fermentation (AL‐Joda and Jasim 2021). A set of Triple sugar iron (TSI) test (Lehman 2005), and motility test and iMViC test including SIM (Sulfide(H_2_S)‐Indole‐Motility Test), Methyl red test, Voges‐Proskauer test (MR‐VP) (McDevitt 2009) and Simmon's Citrate Agar test (T. Acharya 2022) were performed on fresh 24 h cultured isolated colony using various standard protocols in duplication for the confirmation. Results were analyzed after incubation at 37°C for 24 h. TSI test results were interpreted for the slant color, butt color (gave insight into carbohydrate fermentation), gas production, and H_2_S production (see Figure 7). TSI was used in the identification of the Enterobacteriaceae. The stabbing culture of semi‐solid SIM media in the test tube was analyzed for the mobility, H_2_S production, and presence of Indole, a by‐product of tryptophan metabolism (see Figure 8). Indole was analyzed by adding 5 drops or 100 μL of Kovac's reagent to the top of the media. MR‐VP test was performed on MRVP liquid media with MR and VP‐A and VP‐B reagents, and color change was observed (see Figure 9). Simmon's Citrate agar test gave insight into the utilization of citrate as the sole source of carbon or not, simply by the color change in the media and growth of the organism on the slant surface (see Figure 8). For each test, a negative control and an appropriate positive control were used.

### 2.4 Antimicrobial Susceptibility Test (AST) With Standardized Disk Diffusion

Standardization is extremely critical in AST to make the result reliable and reproducible, and also for comparative analysis between results of different countries and laboratories. All the methods in AST are sensitive to any variations in parameters like size of inoculum, content and acidity (pH) of agar medium, diffusion rate of the antimicrobial into the agar, agar depth, dryness of the agar surface, incubation time and temperature, growth rate of the bacteria, and reading procedure. Thus, the International CLSI‐M100 guideline (CLSI 2021) for zone diameter interpretation and CLSI M02 (CLSI 2012) were used for method standardization (inoculum, medium, and incubation). Kirby Bauer's Disk Diffusion Method (Bratu et al. 2005) was used to determine the susceptibility or resistance of Gram‐negative bacterial isolates to antibiotics using commercially available antibiotic discs on Mueller–Hinton Agar (MHA). Each isolate was analyzed in triplicate for 16 antibiotics of 7 major classes (Tetracycline, β‐lactam, Carbapenem, Cephalosporin, Phenicols, Quinolones, and Aminoglycoside), which are used in the treatment of Gram‐negative bacteria in hospital settings (see Table SA.1 for a list of antibiotics used and their disk content).

**Figure 10:**
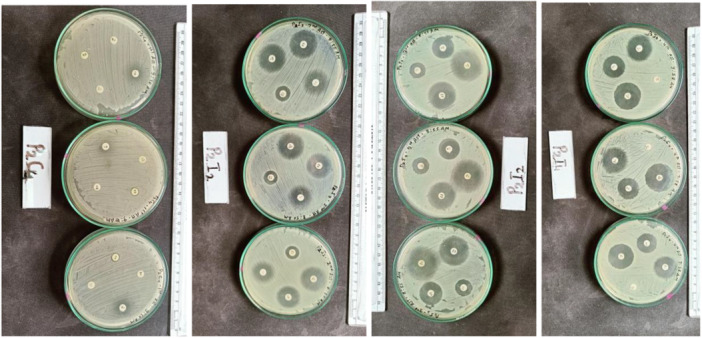
Antimicrobial susceptibility test for all 16 antibiotics.

In the aseptic environment, standard suspensions of the fresh culture of isolates dissolved in 0.85% NaCl adjusted to 0.5 McFarland Standard were prepared. Immediately after standardization, a sterile cotton swab was immersed in the bacterial suspension, and the bacterial isolates were aseptically transferred to the plate to form a lawn culture. MHA plates were prepared with a uniform thickness of 4 mm in the petri plate, which required 20 mL of MHA in 90 mm petriplates (see Appendix S1B of CLSI guidelines M02 for method and various parameters to consider [CLSI 2012]). Four different antibiotic discs were arranged on the surface of one inoculated plate, placed at a distance of > 2.5 cm apart, and the plates were incubated at 37°C for 16–18 h. Appropriate QC strains (ATCC strain) of *S. aureus* ATCC 25923 (BSL 2) as extra checks on test parameters were used (Hudzicki 2009). The results were obtained as shown in Figure 10.

In laboratory settings, results from agar disc diffusion tests are reported qualitatively as Susceptible (S), Intermediate (I), or Resistant (R) based on the zone diameter interpretation chart extracted from CLSI‐M100 guideline (CLSI 2021).

### 2.5 Glycerol Stock Preparation

Sterile liquid LB broth was inoculated with a single colony and incubated for 24 h at 37°C. Seven hundred microliters of the LB culture was added to 700 μL of 40% glycerol (sterile) in a 2 mL Eppendorf tube and gently mixed by pipetting. All relevant information, such as date, researcher, and isolate code, was written on the tube and wrapped in parafilm to prevent accidental opening of the vial. The glycerol stock tube was kept at −20°C. The stocks were produced in pairs: one designated for long‐term storage and the other as a working stock in case of further requirement of tests or process repetition.

### 2.6 Data Analysis

The laboratory data collected were entered into Excel and R for data analysis. Descriptive statistics were used to summarize the data on the spectrum of bacterial pathogens contaminating the meat samples and their AMR rates, MDR prevalence, and multiple antimicrobial resistance (MAR) indices.

## 3 Result

### 3.1 Microbiological Analysis

Upon initial isolation using the spread plate technique, plates exhibiting 20–200 colonies were used for further analysis. Using an integrated approach combining biochemical assays, colony morphology analysis, and Gram staining techniques, the bacterial identities of the isolates were effectively determined.

#### 3.1.1 Gram Staining

Gram staining revealed that all isolates were Gram‐negative (pink reaction). Out of 136 total isolates, most (133) were rod‐shaped, while 3 exhibited a bacilli‐cocci morphology.

#### 3.1.2 Colony Morphology in MacConkey

Among the isolates, 48 did not ferment lactose, whereas 88 displayed lactose fermentation, as evidenced by their pink colonies on MacConkey agar.

#### 3.1.3 Biochemical Tests

Collectively, the biochemical tests provided some insights into the metabolic capabilities of the isolates, paving the way for more specific identification of organisms (see Tables SB.1 and SB.2 for test interpretation for bacterial identification).

A total of 136 isolates were recovered (117 from chicken and 19 from pork sources). The taxonomic distribution of these isolates is summarized in Table 1. The isolates were identified as *E. coli* (46, 33.82%), *Klebsiella* spp. (7, 5.51%), *Shigella* spp. (16, 11.76%), *Salmonella* spp. (5, 3.68%), *Citrobacter* spp. (12, 8.82%), *Proteus* spp. (12, 8.82%), *Serratia* spp. (2, 1.47%), *Yersinia* spp. (12, 8.82%), *Enterobacter* spp. (3, 2.21%), and *Plesiomonas* spp. (7, 5.15%).

**Table 1:** Distribution of microbes isolated in meat samples.

Bacteria	Chicken		Pig		Total *N*	Total %
	*N*	%	*N*	%		
*Citrobacter* spp.	15	8.55%	3	10.53%	18	8.82%
*Enterobacter* spp.	4.5	2.56%	0	0.00%	4.5	2.21%
*Escherichia* spp.	58.5	33.33%	10.5	36.84%	69	33.82%
*Klebsiella* spp.	7.5	4.27%	3	10.53%	10.5	5.15%
Other	12	6.84%	0	0.00%	12	5.88%
*Plesiomonas* spp.	6	3.42%	4.5	15.79%	10.5	5.15%
*Proteus* spp.	18	10.26%	0	0.00%	18	8.82%
*Salmonella* spp.	6	3.42%	1.5	5.26%	7.5	3.68%
*Serratia* spp.	3	1.71%	0	0.00%	3	1.47%
*Shigella* spp.	22.5	12.82%	1.5	5.26%	24	11.76%
Unidentified	6	3.42%	3	10.53%	9	4.41%
*Yersinia* spp.	16.5	9.40%	1.5	5.26%	18	8.82%
Grand total	175.5	100.00%	28.5	100.00%	204	100.00%

### 3.2 ASTs

The majority of the Enterobacteriaceae isolates exhibited resistance to several selected antibiotics. These included ampicillin (82.22%), amoxicillin (71.14%), nalidixic acid (68.21%), and tetracycline (52.86%). In contrast, certain antibiotics maintained high susceptibility rates against these isolates. Amikacin had a 100% susceptibility rate, gentamicin 83.97%, imipenem 96.89%, and meropenem 98.83%. See Table 2. Similar patterns were observed for isolates from both chicken and pig meat samples (see Table 3). But some deviations were observed in resistance to ofloxacin, chloramphenicol, tetracycline, ceftazidime, streptomycin, imipenem, and ciprofloxacin.

**Table 2:** Antibiotics susceptibility pattern.

Antibiotics	I	R	S
Amikacin	0%	0%	100.00%
Amoxicillin	11.08%	71.14%	17.78%
Ampicillin	4.44%	82.22%	13.33%
Ceftazidim	21.39%	27.17%	51.45%
Chloramphenicol	3.43%	28.29%	68.29%
Ciprofloxacin	27.41%	38.78%	33.82%
Gentamycin	1.74%	14.29%	83.97%
Imipenem	2.80%	0.31%	96.89%
Meropenem	0.58%	0.58%	98.83%
Nalidixic acid	4.63%	68.21%	27.16%
Neomycin	10.20%	11.08%	78.72%
Ofloxacin	10.00%	24.29%	65.71%
Piperacillin Tazobactum	1.32%	0%	98.68%
Streptomycin	4.18%	20.56%	75.26%
Tetracycline	12.00%	52.86%	35.14%

**Table 3:** Resistance pattern for 16 different antibiotics tested for both chicken and pork meat samples.

Antibiotics	Chicken			Pig		
	R%	S%	I%	R%	S%	I%
Ampicillin	82.22%	13.33%	4.44%	74.07%	0.00%	25.93%
Amikacin	0.00%	100.00%	0.00%	0.00%	100.00%	0.00%
Ofloxacin	24.50%	65.42%	10.09%	5.36%	92.86%	1.79%
Chloramphenicol	28.53%	68.01%	3.46%	5.36%	89.29%	5.36%
Tetracyclin	52.45%	35.45%	12.10%	35.71%	44.64%	19.64%
Ceftazidim	27.65%	50.59%	21.76%	20.00%	80.00%	0.00%
Streptomycin	20.77%	75.00%	4.23%	15.79%	66.67%	17.54%
Gentamycin	14.44%	83.80%	1.76%	12.28%	84.21%	3.51%
Piparacillin Tazobactum	0.00%	99.00%	1.00%	0.00%	98.25%	1.75%
Nalidixic acid	68.63%	26.71%	4.66%	45.61%	38.60%	15.79%
Imipenam	0.31%	96.89%	2.80%	10.53%	78.95%	10.53%
Amoxicillin	71.14%	17.78%	11.08%	80.70%	12.28%	7.02%
Neomycin	11.08%	78.72%	10.20%	8.77%	89.47%	1.75%
Meropenam	0.58%	98.83%	0.58%	0.00%	98.25%	1.75%
Ciprofloxacin	38.78%	33.82%	27.41%	26.32%	57.89%	15.79%
Grand total	26.82%	66.00%	7.18%	19.51%	72.86%	7.64%

On susceptibility pattern analysis in each isolate for the MDR pattern, out of all organisms tested, 68 were identified as MDR, accounting for 50% of the total (see Table 4).

**Table 4:** MDR frequency table in all isolates.

MDR	Frequency	Relative frequency
Yes	68	50%
No	68	50%
Grand total	136	100.00%

Furthermore, when MDR patterns were observed for each isolate, there were 34 (73.9%) MDR *E. coli* in all samples, 8 MDR *Shigella*, 1 (14.28%) MDR *K. pneumoniae*, 7 MDR *Yersinia*, and 5 MDR *Citrobacter*. This corroborates to 73.9% of all isolated *E. coli* being multidrug resistant, 14.28% of the isolated *Klebsiella*, 50% of *Shigella* spp., 58.33% of the isolated *Yersinia*, and 41.67% of the isolated *Citrobacter* to be multidrug resistant (see Table 5).

**Table 5:** Frequencies of MDR among different isolated bacteria.

Microbe identified	*n* (MDR)	*n* (non‐MDR)	% MDR	% Non‐MDR
*Escherichia* spp.	34	12	73.91	26.09
*Shigella* spp.	8	8	50	50
*Citrobacter* spp.	5	7	41.67	58.33
*Enterobacter* spp.	0	3	0	100
*Klebsiella* spp.	1	6	14.28	85.71
*Plesiomonas* spp.	1	6	14.28	85.71
*Proteus* spp.	4	8	33.33	66.66
*Salmonella* spp.	1	4	20	80
*Serratia* spp.	1	1	50	50
*Yersinia* spp.	7	5	58.33	41.67

**Figure 11:**
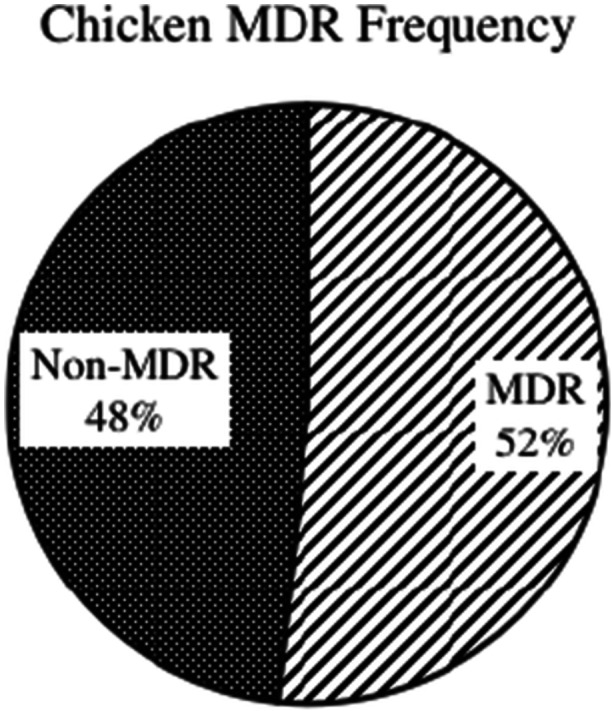
Chicken MDR pattern.

**Figure 12:**
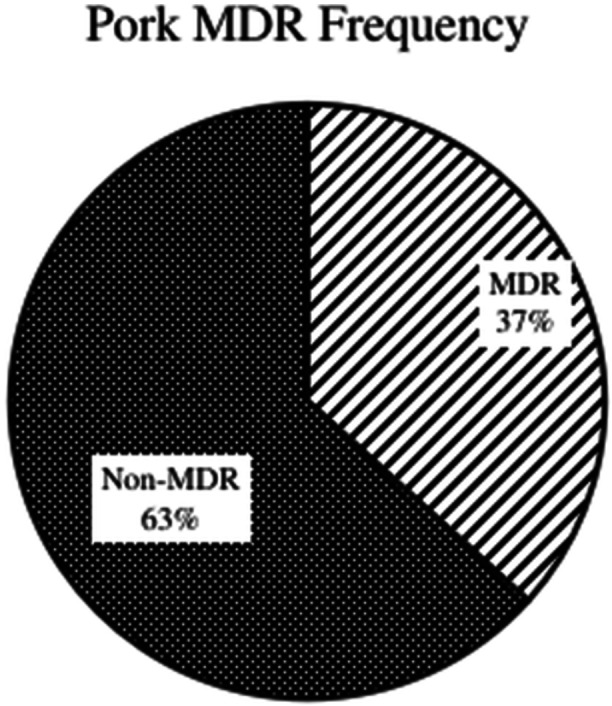
Pork MDR pattern.

Overall, 51.72% isolates from chicken meat were found to be MDR, and 36.84% were found for pork raw meat (see Figures 11 and 12).

Overall, 50% isolates had multiple antibiotic resistance index (MARI) values ≥ 2 (see Table 6).

**Table 6:** Mean distribution of the MAR index of isolates between chicken and pork meat.

Meat	MAR ≥ 2	MAR < 2	Mean MAR
Chicken	51.28%	48.72%	0.25
Pig	42.11%	57.89%	0.198
Grand total	50.00%	50.00%	0.241

## 4 Discussion

### 4.1 Microbiological Analysis

Although the sample size (*n* = 13) is relatively small, the recovery of 136 distinct Gram‐negative isolates, including significant pathogens like *E. coli* (33.82%) and *Shigella* spp. (11.76%), provides a high‐resolution phenotypic map of the resistance burden in the Kathmandu Valley. In our study, among the 13 identified bacterial strains, *E. coli* was the predominant species, accounting for 33.824% of the total. This was followed by *Shigella* spp. at 11.765%. Other significant species were *Yersinia* spp., *Citrobacter*, and *Proteus* spp., each accounting for 8.8%. *Klebsiella* spp. and *Plesiomonas* spp. accounted for 5.1% each, while the remaining species comprised other bacteria (see Table 1). In other recent studies conducted in Nepal, specifically in the Banepa and Dhulikhel Municipalities, meat samples predominantly contained microorganisms such as *E. coli* (62.8%), *Enterococcus* (14.1%), and *K. pneumoniae* (11.5%) (Madhup et al. 2021). This observation is consistent with another study that identified pathogens such as *E. coli*, *Klebsiella*, *Citrobacter*, *Staphylococcus*, and *Salmonella* in raw meat samples (Saud et al. 2019). Expanding on this, another study involving 38 meat samples from Nepal detected 103 Gram‐negative bacteria. This included species such as *Citrobacter* spp. (44.7%), *Salmonella* spp. (26.2%), *Proteus* spp. (18.4%), *E. coli* (4.8%), *Shigella* spp. (3.9%), *Pseudomonas* spp. (1.9%), and *Klebsiella* spp. (1.0%) (Shrestha et al. 2017). The high prevalence of *E. coli* and *Shigella* spp. in our study is attributable to the intestinal and cloacal samples. The results suggest that micro‐organisms such as *E. coli*, *Shigella*, and so forth indicate a high risk of food‐borne infection when raw meat dishes contaminated with feces are consumed or when meat products are handled.

In chicken meat samples, the prevalence of Escherichia spp. is the highest, and so is the case with pork meat samples. It is followed by *Shigella* spp., *Proteus* spp., *Yersinia* spp., *Citrobacter* spp., *Klebsiella* spp., *Salmonella* spp., and so forth. In the pork meat sample, the following organisms are found: *Plesiomonas* spp., *Klebsiella* spp., *Citrobacter* spp., *Shigella* spp., *Salmonella* spp., and *Yersinia* spp.

### 4.2 ASTs and MDR

Out of the organisms tested for MDR, 68 were identified as MDR, accounting for 50% of the total (see Table 4). The data indicate an even split between multidrug‐resistant organisms and their non‐resistant counterparts. The finding that 50% of isolates were MDR indicates a high frequency of acquired resistance, posing a substantial challenge for clinical treatment options. In addition, the observed 50% MDR rate suggests a high potential for horizontal gene transfer via mobile genetic elements, such as plasmids, which are common in Gram‐negative bacteria and facilitate the spread of resistance.

The prevalence of MDR isolates was found to be 79.6% in chicken meat samples in the study by Shrestha et al. (2017). Compared to a similar study (Baah et al. 2022) conducted in Ghana in 2022, which reported a 14.9% MDR rate in beef, goat, and chicken samples. Current data, alongside previous findings from Nepal, highlight a critical escalation of AMR in food‐derived pathogens, particularly in the Kathmandu Valley (Magiorakos et al. 2012; Saud et al. 2019). It further underscores the need for continuous surveillance and updated research to better understand the evolving dynamics of AMR.

Furthermore, on comparative analysis of chicken and pork meat samples with the MDR bacterial isolates, the incidence of MDR isolates was higher among the chicken sample than in the pork meat sample, see Figures 11 and 12. No other studies have been conducted to compare chicken and pork meat in Nepal. Statistical analysis indicated a weak correlation between the resistance profiles of isolates from chicken and pork samples.

For 73.9% of all isolated *E. coli* being multidrug resistant, 14.28% of the isolated *Klebsiella*, 58.33% of the isolated *Yersinia*, and 41.67% of the isolated *Citrobacter* being multidrug resistant, there is a high risk of food‐borne infection and contact‐exposure transmission to slaughterhouse workers by pathogenic microbes, calling for stringent treatment options.

The multidrug‐resistant bacterial isolates identified in this study may be due to the indiscriminate use of antimicrobial agents in poultry and pork. The higher MDR rate in chicken (51.72%) versus pork (36.84%) likely reflects the intensified production of the poultry sector to meet nearly half of the national meat demand, which often leads to overcrowding and higher reliance on prophylactic antibiotics.

In the context of Nepal, tetracycline, enrofloxacin, neomycin‐doxycycline, levofloxacin, colistin, and tylosin emerge as the predominant antibiotics consumed. Moreover, there's an alarmingly high rate of inappropriate prescription, especially with antibiotics like ampicillin, amoxicillin, ceftriaxone, and gentamicin, as highlighted by (K. P. Acharya and Wilson 2019). Correlating this with the findings from our study reveals the high frequency of resistance we observed, especially against beta‐lactam, chloramphenicol, quinolones, and tetracycline classes, can be associated with the use of these antibiotics (see Table 2). The frequent, sometimes unsupervised consumption of these top antibiotics potentially leads to a suitable environment for the emergence of antibiotic‐resistant strains. This is supported by our research findings, which indicate a pressing need for enhanced antibiotic stewardship and more stringent prescribing guidelines in Nepal to mitigate the escalating threat of antibiotic resistance.

## 5 Conclusion and Recommendation

AMR is a pressing global health crisis with profound implications for healthcare systems and patient safety. We conducted a cross‐sectional pilot study in Kathmandu, collecting chicken and pork samples from various sources and subjecting them to rigorous analysis for resistance to 16 antibiotics across six major classes. The results showed a disconcerting trend of antibiotic resistance, with a noteworthy proportion, that is, 50% of bacteria exhibiting MDR. There is an urgent need for stringent regulatory actions to reform unregulated antibiotic sales and implement evidence‐based prescribing guidelines for veterinarians. Notably, when comparing chicken and pork meat samples, a higher prevalence of MDR isolates was observed in chicken (51.72%) than in pork (36.84%), suggesting that MDR is more prevalent in chicken meat. Additionally, the presence of MDR pathogenic organisms, such as *E. coli*, *Shigella*, *K. pneumoniae*, *Salmonella*, *Yersinia*, and *Citrobacter*, raises particularly alarming concerns, especially for individuals working in slaughterhouses who face an elevated risk of infection from these drug‐resistant pathogens. Furthermore, educational initiatives for slaughterhouse workers on hygiene and for consumers on the risks of handling raw meat are essential to mitigate the risk of zoonotic transmission. This study adds to the limited existing knowledge of AMR in consumable meat from food animals (chicken and pork) in the Kathmandu Valley. Also, it highlights the urgent need for responsible antibiotic use in livestock to mitigate the emergence and spread of antibiotic‐resistant bacteria.

### 5.1 Limitations and Future Research

The major limitation of this research is that it involves only chicken and pork meat samples, and the inferred antimicrobial susceptibility patterns may not be representative of foods of other animal origins. Additionally, the sample size is small, which may not adequately capture the full spectrum of resistance in chicken and pork meats, potentially affecting the robustness of comparisons between the two.

Future research should employ longitudinal designs and larger, randomized sample sizes across all seven provinces of Nepal. Additionally, surveillance should expand to include buffalo meat, which accounts for 33% of national consumption, and should utilize molecular techniques (e.g., PCR or WGS) to identify specific resistance genes.

## Author Contributions

**Sushma Paudel:** conceptualization (lead), formal analysis (lead), investigation (lead), writing – original draft (lead), writing – review and editing (equal). **Sumitra Tamang:** investigation (lead), data curation (supporting), conceptualization (supporting). writing – review and editing (equal). **Preety Singh:** investigation (supporting), data curation (supporting), conceptualization (supporting). **Rishav Das:** investigation (supporting), data curation (supporting), conceptualization (supporting). **Sushmita Paudel:** writing – review and editing (lead). Data analysis (supporting). **Astha Shakya:** conceptualization (lead), methodology (supporting), writing – review and editing (equal).

## Funding

The authors have nothing to report.

## Ethics Statement

The authors have nothing to report.

## Conflicts of Interest

The authors declare no conflicts of interest.

## Supporting information

supplimentary 1 tables.
