# Supplementary material for: A Pilot Study on the Prevalence and Characterization of Multidrug‐Resistant Gram‐Negative Bacteria in Chicken and Pork Meat Around Kathmandu District, Nepal
Source: Microbiologyopen. 2026 Mar 27;15(2):e70275. doi: 10.1002/mbo3.70275 (PMC13140625; doi:10.1002/mbo3.70275)
Supplement: Supplementary file 1 — supplimentary 1 tables. [file MBO3-15-e70275-s001.docx]

**Appendix: Equipments, Materials and Media Used**

The commercially available disk of antibiotics with the specified disk content were utilized to carry out AST. Following 16 antibiotics from 6 different classes were selected. Their interpretive zone of inhibition diameter in mm is also shown in the table of Table A.1 (CLSI, 2021).

Table A.1: Test/Report Group Antimicrobial Agent for Enterobacterales; Disk Content; Interpretive Categories and Zone Diameter Breakpoints, nearest whole mm

| **SN** | **class** | **Antimicrobial agent** | **Disk content** | **S** | **I** | **R** |
| --- | --- | --- | --- | --- | --- | --- |
| 1 | Aminoglycosides | Gentamicin | 10 mcg | >=15 | 13-14 | <=12 |
| 2 | Aminoglycosides | Amikacin | 30 mcg | >=17 | 15-16 | <=14 |
| 3 | Aminoglycosides | Streptomycin | 10 mcg | >=15 | 12-14 | <=11 |
| 4 | Aminoglycosides | Neomycin | 10 mcg | >=17 | 13-16 | <=12 |
| 5 | B-lactam combination agent | Pipiracillin-tazobactam | 100/10 mcg | >=21 | 15-19 | <=14 |
| 6 | Carbapenam | Imipenem | 10 mcg | >=23 | 20-22 | <=19 |
| 7 | Carbapenam | Meropenem | 10 mcg | >=23 | 20-22 | <=19 |
| 8 | Cephems | Ceftazidim | 30 mcg | >=21 | 18-20 | <=17 |
| 9 | penicillins | Ampicillin | 10 mcg | >=17 | 14-16 | <=13 |
| 10 | penicillins | Amoxicillin | 10 mcg | >=17 | 14-16 | <=13 |
| 11 | Phenicols | Chloramphenicol | 30 mcg | >=18 | 13-17 | <=12 |
| 12 | Quinolones and flourquinolones | Ciprofloxacin | 5 mcg | >=26 | 22-25 | <=21 |
| 13 | Quinolones and flourquinolones | Nalidixic Acid | 30 mcg | >=19 | 14-18 | <=13 |
| 14 | Quinolones and flourquinolones | Ofloxacin | 5 mcg | >=16 | 13-15 | <=12 |
| 15 | Tetracycline | Tetracycline | 30 mcg | >=15 | 12-14 | <=11 |
| 16 |  | Ceftazidim +calvulanic acid | 30/10 mcg |  |  |  |

Source: CLSI (2021)

## **Appendix: Test interpretation chart and Statistical analysis**

Table B.1: Classification of Enterobacteriaceae based on IMViC test

| Lactose | ++00 | 00++ | 0+0+ | ++0+ | 0+00 |
| --- | --- | --- | --- | --- | --- |
| Rapid | *Escherichia, Yersinia* V(+/0),+00, **Plesiomonas shigelloides* | *Klebsiella, Klebsiella oxytoca* (+0++), *Raoultella* (V0++), *Enterobacter,* Cronobacter | *Citrobacter, Yersinia* V(+/0),+00 |  |  |
| Late | *Escherichia* | *Hafnia alveri* (00+0), *Klebsiella pneumonia, Enterobacter, Serratia* | *Citrobacter, Salmonella* | *Citrobacter* | *Shigella sonnei, Klebsiella pneumoniae subs. ozaenae, Klebsiella pneumo- niae subs. rbinoscleromatis, Proteus penneri* |
| Negative | *Eddwardsiella, Proteus vulgaris, Morganella morganii, *Plesiomonas* |  | *Salmonella, Salmonella enterica serotype Typhi*(0+00), *Proteus mirabilis* 0+V(+/0)v(+/0) | *Providencia* | *Shigella, Yersinia, Salmonella en- terica serotype Typhi*(0+00), Proteus mirabilis 0+V(+/0)v(+/0) |

’0’= negative, ’+’= Positive; * =Oxidase Positive

The order of test is Indole, MR, VP and Citrate. e.g. 00++ = I -, MR-, VP+, C+

Sourced: de la Maza et al. (2020)

Table B.2: TSI test result interpretation for Enterobacteriaceae

| A/AG | A/A G H2 S+ | ALK/A | ALK/A G | ALK/A G H2S+ | ALK/A H2 S+ |
| --- | --- | --- | --- | --- | --- |
| *Citrobacter* spp.  *Cronobacter*  *Enterobacter,*   1. *coli,*   *Klebsiella* spp.  *Enterobacter Pantoea* (IMViC=00++)  *Pluralibacter gergoviae* (V+++) Plesiomonas shigelloides (no gas) Yersinia spp. (gas variable) | *Citrobacter* spp.  *proteus vulgaris* | *Escherichia coli*,  *Klebsiella pneumoniae* subsp. rhinoscleromatis,  *Yersinia* spp.,  *Proteus penneri*  *Morganella*  *Serratia* spp.  *Providencia* spp.  *Shigella* spp. | *Escherichia coli,*  *Salmonella enterica* serovar paratyphi,  *Enterobacter* spp.,  *Hafnia, Klebsiella* spp.,  *Serratia* spp., *Yersinia kristensenii,*  *Citrobacter* spp.,  *Providencia alcalifaciens,*  *Proteus myxofaciens* | *Citrobacter* spp.,  *Salmonella* serovars other than Typhi and Paratyphi,  *Edwardsiella tarda,*  *Proteus mirabilis* | *Salmonella enterica* serotype Typhi |

## **Antimicrobial Susceptibility Tests(AST)**

Table A.4: AST Data observed 1

| **Microbe** | **Isolate** | **AMP mean** | | **AK mean** | | **OF mean** | | **C mean** | | **TE mean** | | **CAZ mean** | |
| --- | --- | --- | --- | --- | --- | --- | --- | --- | --- | --- | --- | --- | --- |
| Escherichia spp. | S1C1 | 0.0 | R | 22.0 | S | 10.0 | R | 0.0 | R | 0.0 | R |  |  |
| Shigella spp. | S1C2 | 17.7 | S | 27.0 | S | 24.3 | S | 28.3 | S | 24.7 | S |  |  |
| Escherichia spp. | S1C3 | 0.0 | R | 25.3 | S | 12.3 | I | 3.7 | R | 9.0 | R |  |  |
| Escherichia spp. | S1C4 | 0.0 | R | 27.0 | S | 0.0 | R | 0.0 | R | 0.0 | R |  |  |
| Shigella spp. | S1C5 | 0.0 | R | 26.7 | S | 29.3 | S | 30.0 | S | 9.7 | R |  |  |
| Escherichia spp. | S1C6 | 0.0 | R | 25.0 | S | 10.3 | R | 0.0 | R | 9.0 | R |  |  |
| Yersinia spp. | S1C7 | 15.0 | I | 26.0 | S | 24.3 | S | 30.3 | S | 0.0 | R |  |  |
| Yersinia spp. | S1C8 | 0.0 | R | 26.7 | S | 8.0 | R | 2.7 | R | 0.0 | R |  |  |
| Citrobacter spp. | S1I1 | 13.0 | R | 24.0 | S | 22.0 | S | 28.0 | S | 23.3 | S |  |  |
| Shigella spp. | S1I2 | 7.0 | R | 23.0 | S | 15.0 | I | 9.3 | R | 12.0 | I |  |  |
| Escherichia spp. | S2C1 | 0.0 | R | 22.3 | S | 10.7 | R | 11.7 | R | 10.7 | R |  |  |
| Escherichia spp. | S2C2 | 11.3 | R | 24.0 | S | 9.3 | R | 0.0 | R | 10.3 | R |  |  |
| Morganella spp. | S2C3 | 13.3 | I | 21.0 | S | 15.7 | I | 13.3 | I | 0.0 | R |  |  |
| Escherichia spp. | S2C4 | 8.0 | R | 21.7 | S | 8.0 | R | 0.0 | R | 7.7 | R |  |  |
| Escherichia spp. | S2C5 | 5.3 | R | 25.7 | S | 8.0 | R | 0.0 | R | 8.0 | R |  |  |
| Escherichia spp. | S2C6 | 9.7 | R | 23.7 | S | 9.7 | R | 0.0 | R | 9.0 | R |  |  |
| Escherichia spp. | S2C7 | 9.0 | R | 19.7 | S | 9.0 | R | 0.0 | R | 8.7 | R |  |  |
| Escherichia spp. | S2i2 | 3.3 | R | 20.3 | S | 13.3 | I | 3.3 | R | 2.7 | R |  |  |
| Escherichia spp. | S2i3 | 0.0 | R | 23.7 | S | 12.0 | R | 0.0 | R | 0.0 | R |  |  |
| Other | S3C1 | 13.0 | R |  |  | 23.7 | S | 30.0 | S | 15.0 | S |  |  |
| Escherichia spp. | S3C2 | 0.0 | R |  |  | 10.3 | R | 22.7 | S | 0.0 | R |  |  |
| Serratia spp. | S3C3 | 8.3 | R |  |  | 24.0 | S | 28.3 | S | 27.3 | S |  |  |
| Escherichia spp. | S3C4 | 0.0 | R |  |  | 9.0 | R | 26.0 | S | 0.0 | R |  |  |
| Unidentified | S3C5 | 0.0 | R |  |  | 25.3 | S | 34.7 | S | 16.7 | S |  |  |
| Unidentified | S3C6 | 11.3 | R |  |  | 22.0 | S | 23.3 | S | 14.0 | I |  |  |
| Other | S3C7 | 0.0 | R |  |  | 14.3 | I | 20.3 | S | 14.0 | I |  |  |
| Salmonella spp. | S3C8 | 0.0 | R |  |  | 15.7 | I | 21.7 | S | 14.0 | I |  |  |
| Escherichia spp. | S3C9 | 0.0 | R |  |  | 19.3 | S | 20.0 | S | 20.7 | S |  |  |
| Shigella spp. | S3C10 | 12.0 | R |  |  | 25.0 | S | 32.7 | S | 29.0 | S |  |  |
| Other | S3C11 | 0.0 | R |  |  | 24.7 | S | 27.0 | S | 26.0 | S |  |  |
| Salmonella spp. | S3C12 | 0.0 | R |  |  | 26.7 | S | 37.0 | S | 15.7 | S |  |  |
| Shigella spp. | S3C13 | 0.0 | R |  |  | 23.0 | S | 31.7 | S | 22.0 | S |  |  |
| Citrobacter spp. | S3C14 | 0.0 | R |  |  | 8.7 | R | 26.7 | S | 3.7 | R |  |  |
| Plesiomonas spp. | S3C16 | 11.7 | R |  |  | 23.7 | S | 32.3 | S | 30.0 | S |  |  |
| Escherichia spp. | S3I1 | 0.0 | R |  |  | 14.7 | I | 25.3 | S | 10.3 | R |  |  |
| Shigella spp. | S3I2 | 0.0 | R |  |  | 23.0 | S | 31.7 | S | 18.0 | S |  |  |
| Escherichia spp. | S3I3 | 0.0 | R |  |  | 9.3 | R | 22.3 | S | 5.7 | R |  |  |
| Shigella spp. | S3I4 | 16.7 | I |  |  | 24.3 | S | 30.0 | S | 28.3 | S |  |  |
| Escherichia spp. | S3I5 | 20.0 | S | 21.7 | S | 30.3 | S | 28.3 | S | 22.0 | S |  |  |
| Citrobacter spp. | S3I6 | 20.0 | S |  |  | 30.0 | S | 22.7 | S | 24.7 | S |  |  |
| Yersinia spp. | S3I7 | 18.0 | S |  |  | 26.0 | S | 33.0 | S | 31.0 | S |  |  |
| Shigella spp. | S4C1 | 0.0 | R | 25.3 | S | 16.0 | S | 3.0 | R | 8.0 | R |  |  |
| Yersinia spp. | S4C2 | 0.0 | R | 22.5 | S | 0.0 | R | 0.0 | R | 0.0 | R |  |  |
| Shigella spp. | S4C3 | 0.0 | R | 26.0 | S | 14.0 | I | 3.0 | R | 8.0 | R |  |  |
| Shigella spp. | S4C4 | 0.0 | R | 25.3 | S | 14.0 | I | 0.0 | R | 0.0 | R |  |  |
| Yersinia spp. | S4C5 | 0.0 | R | 24.0 | S | 14.3 | I | 0.0 | R | 6.0 | R |  |  |
| Shigella spp. | S4I1 | 0.0 | R | 24.3 | S | 14.7 | I | 3.0 | R | 8.3 | R |  |  |
| Escherichia spp. | S4I2 | 0.0 | R | 24.7 | S | 24.3 | S | 10.3 | R | 2.7 | R |  |  |
| Escherichia spp. | S4I3 | 0.0 | R | 22.3 | S | 24.7 | S | 0.0 | R | 5.3 | R |  |  |
| Escherichia spp. | S4I4 | 0.0 | R | 24.3 | S | 9.3 | R | 25.3 | S | 23.3 | S |  |  |
| Yersinia spp. | S4I5 | 25.3 | S | 29.3 | S | 34.3 | S | 32.0 | S | 30.7 | S |  |  |
| Escherichia spp. | S5C1 | 0.0 | R | 22.3 | S | 22.3 | S | 0.0 | R | 0.0 | R | 24.7 | S |
| Shigella spp. | S5C2 | 0.0 | R | 22.0 | S | 23.0 | S | 27.7 | S | 12.0 | I | 25.7 | S |
| Escherichia spp. | S5C3 | 0.0 | R | 25.0 | S | 0.0 | R | 0.0 | R | 0.0 | R | 21.3 | S |
| Escherichia spp. | S5C4 | 0.0 | R | 21.3 | S | 22.7 | S | 25.7 | S | 9.7 | R | 25.3 | S |
| Proteus spp. | S5C5 | 0.0 | R |  | R |  | R |  | R |  | R | 25.3 | S |
| Escherichia spp. | S5I1 | 0.0 | R | 21.3 | S | 20.7 | S | 11.3 | R | 11.0 | R | 25.3 | S |
| Escherichia spp. | S5I2 | 0.0 | R | 22.7 | S | 31.7 | S | 27.0 | S | 12.0 | I | 25.7 | S |
| Proteus spp. | S5I3 | 0.0 | R | 25.0 | S | 22.7 | S | 30.0 | S | 12.0 | I | 25.0 | S |
| Yersinia spp. | S5I4 | 0.0 | R | 21.7 | S | 23.0 | S | 26.7 | S | 8.3 | R | 26.7 | S |
| Proteus spp. | S5I5 | 0.0 | R | 24.3 | S | 22.3 | S | 13.0 | I | 7.0 | R | 21.3 | S |
| Escherichia spp. | S6C1 | 0.0 | R | 21.7 | S | 10.7 | R | 0.0 | R | 9.3 | R |  |  |
| Unidentified | S6C2 | 0.0 | R | 22.7 | S | 23.7 | S | 31.7 | S | 16.7 | S | 26.0 | S |
| Escherichia spp. | S6C3 | 0.0 | R | 22.7 | S | 10.7 | R | 10.7 | R | 3.0 | R |  |  |
| Citrobacter spp. | S6C4 | 0.0 | R | 25.7 | S | 0.0 | R | 27.0 | S | 0.0 | R |  |  |
| Unidentified | S6C5 | 0.0 | R | 23.3 | S | 17.7 | S | 28.0 | S | 17.0 | S |  |  |
| Escherichia spp. | S6I1 | 16.3 | I | 23.0 | S | 21.7 | S | 0.0 | R | 13.3 | I |  |  |
| Salmonella spp. | S6I2 | 15.0 | I | 27.7 | S | 25.3 | S | 31.7 | S | 14.3 | I |  |  |
| Citrobacter spp. | S6I3 | 16.7 | I | 26.0 | S | 28.0 | S | 27.7 | S | 13.3 | I |  |  |
| Escherichia spp. | S6I4 | 0.0 | R | 22.7 | S | 0.0 | R | 4.0 | R | 6.0 | R |  |  |
| Yersinia spp. | S6I5 | 11.3 | R | 21.0 | S | 0.0 | R | 28.0 | S | 10.3 | R |  |  |
| Enterobacter spp. | S7C1 | 0.0 | R | 22.3 | S | 26.0 | S | 26.3 | S | 22.7 | S | 25.7 | S |
| Klebsiella spp. | S7C2 | 0.0 | R | 22.7 | S | 29.0 | S | 26.7 | S | 24.3 | S | 25.3 | S |
| Proteus spp. | S7C3 | 0.0 | R | 24.7 | S | 21.3 | S | 18.7 | S | 15.3 | S | 5.0 | R |
| Escherichia spp. | S7C4 | 0.0 | R | 21.7 | S | 10.7 | R | 29.7 | S | 10.3 | R | 28.7 | S |
| Shigella spp. | S7C5 | 0.0 | R | 20.0 | S | 12.7 | I | 26.7 | S | 7.7 | R | 22.7 | S |
| Proteus spp. | S7I1 | 0.0 | R | 23.3 | S | 22.7 | S | 32.0 | S | 14.3 | I | 27.0 | S |
| Klebsiella spp. | S7I2 | 0.0 | R | 22.7 | S | 28.3 | S | 25.0 | S | 22.0 | S | 25.7 | S |
| Citrobacter spp. | S7I3 | 0.0 | R | 22.3 | S | 24.7 | S | 20.7 | S | 8.7 | R | 21.7 | S |
| Plesiomonas spp. | S7I4 | 0.0 | R | 26.7 | S | 24.3 | S | 22.0 | S | 24.0 | S | 21.3 | S |
| Escherichia spp. | S7I5 | 0.0 | R | 23.3 | S | 13.3 | I | 0.0 | R | 0.0 | R | 25.7 | S |
| Other | S8C1 | 0.0 | R | 22.7 | S | 15.0 | I | 26.7 | S | 0.0 | R | 29.3 | S |
| Escherichia spp. | S8C2 | 0.0 | R | 25.3 | S | 0.0 | R | 24.0 | S | 3.0 | R | 19.7 | I |
| Escherichia spp. | S8C3 | 0.0 | R | 26.0 | S | 18.5 | S | 23.5 | S | 0.0 | R | 0.0 | R |
| Shigella spp. | S8C4 | 0.0 | R | 27.0 | S | 22.7 | S | 18.0 | S | 9.3 | R | 7.3 | R |
| Yersinia spp. | S8C5 | 0.0 | R | 23.3 | S | 0.0 | R | 0.0 | R | 0.0 | R | 0.0 | R |
| Serratia spp. | S8I1 | 0.0 | R | 23.7 | S | 21.0 | S | 24.7 | S | 8.3 | R | 26.7 | S |
| Klebsiella spp. | S8I2 | 0.0 | R | 23.0 | S | 28.7 | S | 25.0 | S | 19.3 | S | 22.0 | S |
| Escherichia spp. | S8I3 | 0.0 | R | 26.0 | S | 20.3 | S | 0.0 | R | 10.0 | R | 0.0 | R |
| Proteus spp. | S8I4 | 0.0 | R | 36.0 | S | 34.5 | S | 28.5 | S | 21.0 | S | 18.7 | I |
| Plesiomonas spp. | S8I5 | 0.0 | R | 23.5 | S | 27.5 | S | 26.0 | S | 19.0 | S | 22.7 | S |
| Klebsiella spp. | S9C1 | 0.0 | R | 24.0 | S | 22.7 | S | 0.0 | R | 9.7 | R | 5.3 | R |
| Plesiomonas spp. | S9C2 | 0.0 | R | 24.7 | S | 29.0 | S | 28.3 | S | 24.3 | S | 6.7 | R |
| Pantoea spp. | S9C3 | 0.0 | R | 30.7 | S | 36.3 | S | 33.0 | S | 29.7 | S | 26.0 | S |
| Proteus spp. | S9C4 | 0.0 | R | 23.3 | S | 22.3 | S | 25.0 | S | 24.0 | S | 0.0 | R |
| Enterobacter spp. | S9C5 | 0.0 | R | 26.3 | S | 31.0 | S | 29.3 | S | 11.7 | I | 7.7 | R |
| Citrobacter spp. | S9I1 | 0.0 | R | 25.7 | S | 29.7 | S | 29.0 | S | 23.3 | S | 24.0 | S |
| Yersinia spp. | S9I2 | 0.0 | R | 28.3 | S | 27.0 | S | 35.7 | S | 18.0 | S | 22.7 | S |
| Citrobacter spp. | S9I3 | 0.0 | R | 28.3 | S | 26.0 | S | 31.0 | S | 15.7 | S | 19.7 | I |
| Proteus spp. | S9I4 | 0.0 | R | 26.7 | S | 24.3 | S | 30.3 | S | 19.7 | S | 23.0 | S |
| Klebsiella spp. | S9I5 | 0.0 | R | 25.3 | S | 25.7 | S | 28.3 | S | 7.7 | R | 22.0 | S |
| Other | S10C1 | 0.0 | R | 24.7 | S | 18.7 | S | 29.3 | S | 3.0 | R | 19.7 | I |
| Yersinia spp. | S10C2 | 0.0 | R | 24.3 | S | 15.3 | I | 31.0 | S | 13.7 | I | 15.3 | R |
| Citrobacter spp. | S10C3 | 0.0 | R | 23.0 | S | 16.3 | S | 24.7 | S | 12.7 | I | 0.0 | R |
| Escherichia spp. | S10C4 | 0.0 | R | 20.3 | S | 21.0 | S | 26.7 | S | 12.3 | I | 0.0 | R |
| Escherichia spp. | S10C5 | 0.0 | R | 25.5 | S | 22.0 | S | 29.0 | S | 11.5 | I | 20.3 | I |
| Proteus spp. | S10I1 | 0.0 | R | 22.7 | S | 19.3 | S | 29.3 | S | 10.0 | R | 20.3 | I |
| Citrobacter spp. | S11C1 | 0.0 | R | 26.3 | S | 24.3 | S | 30.0 | S | 14.0 | I | 0.0 | R |
| Proteus spp. | S11C2 | 0.0 | R | 26.0 | S | 23.3 | S | 20.3 | S | 18.3 | S | 17.3 | I |
| Proteus spp. | S11C3 | 0.0 | R | 24.3 | S | 22.0 | S | 27.7 | S | 13.0 | I | 18.0 | I |
| Proteus spp. | S11C4 | 0.0 | R | 26.7 | S | 28.3 | S | 35.3 | S | 17.0 | S | 20.7 | I |
| Enterobacter spp. | S11C5 | 0.0 | R | 22.7 | S | 21.0 | S | 27.0 | S | 0.0 | R | 19.3 | I |
| Pantoea spp. | S11I1 | 0.0 | R | 23.7 | S | 15.7 | I | 13.0 | I | 5.3 | R | 0.0 | R |
| Shigella spp. | S11I2 | 0.0 | R | 23.0 | S | 24.3 | S | 30.7 | S | 16.0 | S | 6.0 | R |
| Escherichia spp. | S11I3 | 0.0 | R | 23.7 | S | 28.3 | S | 30.7 | S | 23.7 | S | 18.0 | I |
| Escherichia spp. | S11I4 | 0.0 | R | 22.3 | S | 10.3 | R | 20.3 | S | 9.0 | R | 18.7 | I |
| Salmonella spp. | S11I5 | 0.0 | R | 24.0 | S | 26.7 | S | 24.0 | S | 15.0 | S | 6.3 | R |
| Escherichia spp. | P1C1 | 0.0 | R | 25.7 | S | 23.7 | S | 21.3 | S | 7.0 | R | 25.0 | S |
| Escherichia spp. | P1C2 | 0.0 | R | 25.7 | S | 16.7 | S | 13.0 | I | 14.7 | I | 26.3 | S |
| Citrobacter spp. | P1C3 | 0.0 | R | 22.7 | S | 24.3 | S | 19.3 | S | 5.3 | R | 11.7 | R |
| Klebsiella spp. | P1C4 | 0.0 | R | 23.7 | S | 29.3 | S | 27.0 | S | 24.7 | S | 26.3 | S |
| Plesiomonas spp. | P1C5 | 0.0 | R | 22.0 | S | 21.3 | S | 30.3 | S | 10.7 | R | 13.0 | R |
| Shigella spp. | P1I1 | 0.0 | R | 22.3 | S | 25.7 | S | 31.0 | S | 14.0 | I | 27.3 | S |
| Unidentified | P1I2 | 0.0 | R | 22.0 | S | 21.0 | S | 27.3 | S | 8.7 | R | 28.3 | S |
| Unidentified | P1I3 | 0.0 | R | 21.0 | S | 26.3 | S | 20.0 | S | 13.0 | I | 22.3 | S |
| Escherichia spp. | P1I4 | 0.0 | R | 22.0 | S | 24.0 | S | 28.5 | S | 9.0 | R | 26.3 | S |
| Klebsiella spp. | P1I5 | 0.0 | R | 21.7 | S | 21.7 | S | 26.7 | S | 0.0 | R | 21.3 | S |
| Salmonella spp. | P2C1 | 0.0 | R | 20.3 | S | 23.7 | S | 29.0 | S | 15.7 | S |  |  |
| Plesiomonas spp. | P2C2 | 0.0 | R | 21.0 | S | 17.0 | S | 30.0 | S | 12.7 | I |  |  |
| Citrobacter spp. | P2C3 | 14.7 | I | 20.3 | S | 30.3 | S | 26.3 | S | 27.3 | S |  |  |
| Escherichia spp. | P2C4 | 0.0 | R | 21.3 | S | 0.0 | R | 12.7 | I | 5.3 | R |  |  |
| Escherichia spp. | P2I1 | 14.3 | I | 22.0 | S | 33.7 | S | 29.0 | S | 24.7 | S |  |  |
| Escherichia spp. | P2I2 | 0.0 | R | 22.7 | S | 25.0 | S | 25.7 | S | 23.0 | S |  |  |
| Escherichia spp. | P2I3 | 13.7 | I | 22.7 | S | 32.7 | S | 29.3 | S | 23.7 | S |  |  |
| Plesiomonas spp. | P2I4 | 0.0 | R | 22.7 | S | 23.3 | S | 33.0 | S | 17.0 | S |  |  |
| Yersinia spp. | P2I5 | 0.0 | R | 21.0 | S | 19.3 | S | 20.0 | S | 14.7 | I |  |  |
| AMP=Ampicillin, AK=Amikacin, OF=Ofloxacin C=Ciprofloxacin, TE=Tetracycline, CAZ=Ceftazidime | | | | | | | | | | | | | |

Table A.5: AST Data observed 2

| **Microbe** | **Isolate** | **CAC mean** | | **S mean** | | **G mean** | | **PIT mean** | | **NA mean** | |
| --- | --- | --- | --- | --- | --- | --- | --- | --- | --- | --- | --- |
| Escherichia spp. | S1C1 | 20.7 |  | 15.7 | S | 13.0 | I | 28.0 | S | 0.0 | R |
| Shigella spp. | S1C2 | 24.0 |  | 23.7 | S | 28.7 | S | 30.0 | S | 24.7 | S |
| Escherichia spp. | S1C3 | 22.3 |  | 0.0 | R | 27.0 | S | 27.0 | S | 0.0 | R |
| Escherichia spp. | S1C4 | 22.3 |  | 19.0 | S | 18.0 | S | 30.3 | S | 0.0 | R |
| Shigella spp. | S1C5 | 26.0 |  | 23.3 | S | 29.3 | S | 31.0 | S | 22.0 | S |
| Escherichia spp. | S1C6 | 22.0 |  | 0.0 | R | 26.7 | S | 30.0 | S | 0.0 | R |
| Yersinia spp. | S1C7 | 21.0 |  | 21.7 | S | 28.0 | S | 21.3 | S | 20.3 | S |
| Yersinia spp. | S1C8 | 21.3 |  | 0.0 | R | 16.0 | S | 26.7 | S | 0.0 | R |
| Citrobacter spp. | S1I1 | 18.0 |  | 19.3 | S | 27.3 | S | 28.7 | S | 0.0 | R |
| Shigella spp. | S1I2 | 19.0 |  | 21.3 | S | 27.3 | S | 36.7 | S | 0.0 | R |
| Escherichia spp. | S2C1 | 22.7 |  | 17.3 | S | 24.7 | S | 29.3 | S | 0.0 | R |
| Escherichia spp. | S2C2 | 21.0 |  | 10.0 | R | 25.3 | S | 28.3 | S | 0.0 | R |
| Other | S2C3 | 19.7 |  | 9.0 | R | 23.0 | S | 21.7 | S | 0.0 | R |
| Escherichia spp. | S2C4 | 20.0 |  | 9.0 | R | 22.7 | S | 25.0 | S | 0.0 | R |
| Escherichia spp. | S2C5 | 19.7 |  | 8.0 | R | 22.3 | S | 23.7 | S | 0.0 | R |
| Escherichia spp. | S2C6 | 20.0 |  | 10.0 | R | 24.3 | S | 25.0 | S | 0.0 | R |
| Escherichia spp. | S2C7 | 19.7 |  | 8.3 | R | 22.7 | S | 24.0 | S | 0.0 | R |
| Escherichia spp. | S2i2 | 16.7 |  | 9.7 | R | 22.3 | S | 24.3 | S | 0.0 | R |
| Escherichia spp. | S2i3 | 17.0 |  | 10.3 | R | 24.3 | S | 22.0 | S | 0.0 | R |
| Other | S3C1 |  |  |  |  |  |  |  |  |  |  |
| Escherichia spp. | S3C2 |  |  |  |  |  |  | 23.7 | S | 0.0 | R |
| Serratia spp. | S3C3 |  |  |  |  |  |  | 29.3 | S | 0.0 | R |
| Escherichia spp. | S3C4 |  |  |  |  |  |  |  |  |  |  |
| Unidentified | S3C5 |  |  |  |  |  |  | 29.3 | S | 9.0 | R |
| Unidentified | S3C6 |  |  |  |  |  |  | 29.3 | S | 8.7 | R |
| Other | S3C7 |  |  |  |  |  |  |  |  |  |  |
| Salmonella spp. | S3C8 |  |  |  |  |  |  |  |  |  |  |
| Escherichia spp. | S3C9 |  |  |  |  |  |  |  |  |  |  |
| Shigella spp. | S3C10 |  |  |  |  |  |  |  |  |  |  |
| Other | S3C11 |  |  |  |  |  |  | 33.7 | S | 9.7 | R |
| Salmonella spp. | S3C12 |  |  |  |  |  |  | 28.7 | S | 9.0 | R |
| Shigella spp. | S3C13 |  |  |  |  |  |  |  |  |  |  |
| Citrobacter spp. | S3C14 |  |  |  |  |  |  |  |  |  |  |
| Plesiomonas spp. | S3C16 |  |  |  |  |  |  |  |  |  |  |
| Escherichia spp. | S3I1 | 20.0 |  |  |  |  |  |  |  | 0.0 | R |
| Shigella spp. | S3I2 | 24.3 |  |  |  |  |  |  |  | 0.0 | R |
| Escherichia spp. | S3I3 | 20.0 |  |  |  |  |  |  |  | 0.0 | R |
| Shigella spp. | S3I4 | 0.0 |  |  |  |  |  |  |  | 6.7 | R |
| Escherichia spp. | S3I5 | 20.0 |  | 20.0 | S | 22.7 | S |  |  | 26.0 | S |
| Citrobacter spp. | S3I6 | 23.7 |  |  |  |  |  |  |  | 27.0 | S |
| Yersinia spp. | S3I7 | 7.3 |  |  |  |  |  |  |  | 11.0 | R |
| Shigella spp. | S4C1 | 22.7 |  | 17.3 | S | 10.0 | R | 32.0 | S | 0.0 | R |
| Yersinia spp. | S4C2 |  |  |  |  |  |  | 28.3 | S | 0.0 | R |
| Shigella spp. | S4C3 | 24.3 |  | 17.3 | S | 10.3 | R | 31.7 | S | 0.0 | R |
| Shigella spp. | S4C4 | 22.7 |  | 17.3 | S | 10.3 | R | 30.3 | S | 0.0 | R |
| Yersinia spp. | S4C5 | 22.3 |  | 15.7 | S | 8.7 | R | 29.3 | S | 0.0 | R |
| Shigella spp. | S4I1 | 22.3 |  | 16.7 | S | 9.7 | R | 29.3 | S | 0.0 | R |
| Escherichia spp. | S4I2 | 24.7 |  | 21.7 | S | 23.3 | S | 30.7 | S | 23.0 | S |
| Escherichia spp. | S4I3 | 23.7 |  | 19.0 | S | 21.7 | S | 30.0 | S | 24.0 | S |
| Escherichia spp. | S4I4 | 22.3 |  | 20.3 | S | 22.3 | S | 26.5 | S | 0.0 | R |
| Yersinia spp. | S4I5 | 12.0 |  | 26.3 | S | 29.0 | S | 28.0 | S | 0.0 | R |
| Escherichia spp. | S5C1 | 18.0 | n | 16.7 | S | 22.7 | S | 23.0 | S | 0.0 | R |
| Shigella spp. | S5C2 | 17.3 | n | 18.7 | S | 23.3 | S | 23.7 | S | 19.3 | S |
| Escherichia spp. | S5C3 | 13.7 | n | 20.3 | S | 12.0 | R | 28.3 | S | 0.0 | R |
| Escherichia spp. | S5C4 | 18.7 | n | 17.0 | S | 23.0 | S | 25.0 | S | 17.7 | I |
| Proteus spp. | S5C5 | 18.7 | n | 16.3 | S | 23.0 | S | 24.0 | S | 20.0 | S |
| Escherichia spp. | S5I1 | 18.7 | n | 17.0 | S | 23.0 | S | 24.7 | S | 7.0 | R |
| Escherichia spp. | S5I2 | 17.3 | n | 19.0 | S | 25.7 | S | 25.0 | S | 22.3 | S |
| Proteus spp. | S5I3 | 18.0 | n | 18.3 | S | 22.0 | S | 24.0 | S | 20.7 | S |
| Yersinia spp. | S5I4 | 18.0 | n | 19.0 | S | 22.0 | S | 28.7 | S | 19.3 | S |
| Proteus spp. | S5I5 | 17.7 | n | 9.0 | R | 24.3 | S | 26.7 | S | 20.7 | S |
| Escherichia spp. | S6C1 | 20.0 |  | 9.0 | R | 20.0 | S | 26.3 | S | 0.0 | R |
| Unidentified | S6C2 | 17.3 | n | 17.0 | S | 22.7 | S | 25.3 | S | 8.7 | R |
| Escherichia spp. | S6C3 | 6.0 |  | 18.3 | S | 10.0 | R | 27.0 | S | 0.0 | R |
| Citrobacter spp. | S6C4 | 16.7 |  | 21.3 | S | 25.7 | S | 28.3 | S | 0.0 | R |
| Unidentified | S6C5 | 15.3 |  | 10.3 | R | 22.0 | S | 26.7 | S | 9.3 | R |
| Escherichia spp. | S6I1 | 19.0 |  | 17.0 | S | 10.3 | R | 26.0 | S | 0.0 | R |
| Salmonella spp. | S6I2 | 20.7 |  | 24.3 | S | 28.7 | S | 30.7 | S | 0.0 | R |
| Citrobacter spp. | S6I3 | 18.0 |  | 21.7 | S | 28.0 | S | 30.3 | S | 23.0 | S |
| Escherichia spp. | S6I4 | 17.7 |  | 14.0 | I | 23.7 | S | 27.0 | S | 0.0 | R |
| Yersinia spp. | S6I5 | 17.3 |  | 15.0 | S | 22.0 | S | 24.7 | S | 0.0 | R |
| Enterobacter spp. | S7C1 | 17.7 | n | 18.0 | S | 22.7 | S | 25.3 | S | 22.7 | S |
| Klebsiella spp. | S7C2 | 16.7 | n | 18.0 | S | 23.0 | S | 25.3 | S | 24.3 | S |
| Proteus spp. | S7C3 | 0.0 | n | 16.0 | S | 22.7 | S | 26.3 | S | 0.0 | R |
| Escherichia spp. | S7C4 | 11.3 | n | 9.0 | R | 12.0 | R | 26.3 | S | 0.0 | R |
| Shigella spp. | S7C5 | 16.0 | n | 16.3 | S | 21.3 | S | 26.0 | S | 0.0 | R |
| Proteus spp. | S7I1 | 17.0 | n | 18.3 | S | 22.3 | S | 27.7 | S | 0.0 | R |
| Klebsiella spp. | S7I2 | 17.7 | n | 19.7 | S | 23.0 | S | 27.7 | S | 24.0 | S |
| Citrobacter spp. | S7I3 | 11.3 | n | 18.3 | S | 23.0 | S | 24.7 | S | 19.7 | S |
| Plesiomonas spp. | S7I4 | 14.0 | n | 20.0 | S | 26.7 | S | 29.7 | S | 3.0 | R |
| Escherichia spp. | S7I5 | 15.3 | n | 10.7 | R | 7.0 | R | 28.7 | S | 0.0 | R |
| Other | S8C1 | 19.7 | n | 11.3 | I | 10.3 | R | 30.3 | S | 0.0 | R |
| Escherichia spp. | S8C2 | 17.3 | n | 19.3 | S | 23.3 | S | 27.0 | S | 0.0 | R |
| Escherichia spp. | S8C3 | 0.0 | n | 19.0 | S | 26.0 | S | 27.3 | S | 0.0 | R |
| Shigella spp. | S8C4 | 0.0 | n | 19.3 | S | 10.7 | R | 29.0 | S | 0.0 | R |
| Yersinia spp. | S8C5 | 0.0 | n | 0.0 | R | 9.7 | R | 26.3 | S | 0.0 | R |
| Serratia spp. | S8I1 | 11.7 | n | 18.3 | S | 9.7 | R | 27.7 | S | 17.0 | I |
| Klebsiella spp. | S8I2 | 18.3 | n | 17.3 | S | 22.0 | S | 28.7 | S | 22.0 | S |
| Escherichia spp. | S8I3 | 0.0 | n | 16.3 | S | 25.3 | S | 28.3 | S | 0.0 | R |
| Proteus spp. | S8I4 | 14.3 | n | 21.7 | S | 30.3 | S | 37.0 | S | 16.3 | I |
| Plesiomonas spp. | S8I5 | 19.0 | n | 16.3 | S | 24.0 | S | 34.0 | S | 25.0 | S |
| Klebsiella spp. | S9C1 | 0.0 | n | 20.0 | S | 23.3 | S | 26.7 | S | 23.0 | S |
| Plesiomonas spp. | S9C2 | 6.3 | n | 20.3 | S | 23.3 | S | 29.3 | S | 18.7 | I |
| Pantoea spp. | S9C3 | 22.7 | n | 25.7 | S | 33.7 | S | 34.7 | S | 26.7 | S |
| Proteus spp. | S9C4 | 0.0 | n | 16.7 | S | 23.0 | S | 31.7 | S | 26.7 | S |
| Enterobacter spp. | S9C5 | 7.0 | n | 21.3 | S | 27.3 | S | 24.0 | S | 21.0 | S |
| Citrobacter spp. | S9I1 | 23.0 | n | 24.7 | S | 31.3 | S | 28.7 | S | 19.0 | S |
| Yersinia spp. | S9I2 | 18.3 | n | 24.7 | S | 29.7 | S | 33.7 | S | 9.0 | R |
| Citrobacter spp. | S9I3 | 13.7 | n | 23.0 | S | 29.3 | S | 23.3 | S | 0.0 | R |
| Proteus spp. | S9I4 | 19.0 | n | 21.5 | S | 31.5 | S | 41.3 | S | 31.3 | S |
| Klebsiella spp. | S9I5 | 19.3 | n | 23.0 | S | 28.3 | S | 29.7 | S | 25.3 | S |
| Other | S10C1 | 18.7 | n | 20.3 | S | 25.0 | S | 28.3 | S | 0.0 | R |
| Yersinia spp. | S10C2 | 13.7 | n | 10.0 | R | 25.3 | S | 29.0 | S | 0.0 | R |
| Citrobacter spp. | S10C3 | 0.0 | n | 20.0 | S | 21.7 | S | 26.3 | S | 0.0 | R |
| Escherichia spp. | S10C4 | 0.0 | n | 18.0 | S | 22.3 | S | 26.7 | S | 18.3 | I |
| Escherichia spp. | S10C5 | 18.3 | n | 19.7 | S | 25.0 | S | 26.7 | S | 21.0 | S |
| Proteus spp. | S10I1 | 18.3 | n | 19.7 | S | 25.0 | S | 27.7 | S | 0.0 | R |
| Citrobacter spp. | S11C1 | 0.0 | n | 22.3 | S | 27.7 | S | 30.3 | S | 0.0 | R |
| Proteus spp. | S11C2 | 15.7 | n | 14.3 | I | 24.7 | S | 33.3 | S | 9.3 | R |
| Proteus spp. | S11C3 | 15.0 | n | 20.0 | S | 25.0 | S | 27.7 | S | 0.0 | R |
| Proteus spp. | S11C4 | 18.3 | n | 17.3 | S | 32.0 | S | 36.0 | S | 8.7 | R |
| Enterobacter spp. | S11C5 | 18.0 | n | 20.3 | S | 24.3 | S | 25.7 | S | 21.7 | S |
| Pantoea spp. | S11I1 | 0.0 | n | 0.0 | R | 17.3 | S | 28.3 | S | 0.0 | R |
| Shigella spp. | S11I2 | 5.7 | n | 12.0 | I | 24.3 | S | 29.7 | S | 6.7 | R |
| Escherichia spp. | S11I3 | 5.3 | n | 18.3 | S | 24.3 | S | 29.7 | S | 11.3 | R |
| Escherichia spp. | S11I4 | 16.7 | n | 18.3 | S | 24.0 | S | 25.3 | S | 0.0 | R |
| Salmonella spp. | S11I5 | 0.0 | n | 17.0 | S | 27.0 | S | 31.7 | S | 11.7 | R |
| Escherichia spp. | P1C1 | 18.7 | n | 19.3 | S | 23.3 | S | 23.0 | S | 19.7 | S |
| Escherichia spp. | P1C2 | 26.3 | n | 0.0 | R | 25.7 | S | 29.3 | S | 0.0 | R |
| Citrobacter spp. | P1C3 | 0.0 | n | 20.0 | S | 25.3 | S | 26.7 | S | 19.0 | S |
| Klebsiella spp. | P1C4 | 19.7 | n | 19.7 | S | 23.3 | S | 26.0 | S | 24.7 | S |
| Plesiomonas spp. | P1C5 | 0.0 | n | 13.0 | I | 23.7 | S | 26.3 | S | 15.3 | I |
| Shigella spp. | P1I1 | 20.3 | n | 10.3 | R | 23.7 | S | 29.3 | S | 21.0 | S |
| Unidentified | P1I2 | 19.7 | n | 14.0 | I | 23.0 | S | 27.0 | S | 0.0 | R |
| Unidentified | P1I3 | 9.3 | n | 18.7 | S | 22.7 | S | 26.0 | S | 17.3 | I |
| Escherichia spp. | P1I4 | 18.7 | n | 12.3 | I | 12.7 | I | 25.7 | S | 9.7 | R |
| Klebsiella spp. | P1I5 | 21.3 | n | 19.3 | S | 22.7 | S | 29.7 | S | 18.3 | I |
| Salmonella spp. | P2C1 | 12.3 |  | 16.7 | S | 21.0 | S | 27.3 | S | 0.0 | R |
| Plesiomonas spp. | P2C2 | 20.0 |  | 18.7 | S | 23.7 | S | 29.3 | S | 0.0 | R |
| Citrobacter spp. | P2C3 | 15.0 |  | 15.0 | S | 20.3 | S | 26.3 | S | 22.3 | S |
| Escherichia spp. | P2C4 | 17.0 |  | 14.7 | I | 10.0 | R | 28.0 | S | 0.0 | R |
| Escherichia spp. | P2I1 | 19.7 |  | 17.0 | S | 23.0 | S | 27.3 | S | 13.0 | R |
| Escherichia spp. | P2I2 | 13.3 |  | 18.0 | S | 21.7 | S | 26.0 | S | 20.7 | S |
| Escherichia spp. | P2I3 | 18.3 |  | 17.3 | S | 24.0 | S | 27.3 | S | 25.0 | S |
| Plesiomonas spp. | P2I4 | 18.7 |  | 18.0 | S | 23.0 | S | 25.3 | S | 0.0 | R |
| Yersinia spp. | P2I5 | 14.3 |  | 10.3 | R | 8.3 | R | 23.0 | S | 0.0 | R |
| CAC=Ceftazidime+calvulanic acid, S=Streptomycin, G=Gentamicin PIT=Piperacillin Tazobactum, NA=Nalidixic acid, | | | | | | | | | | | |
|  |  |  |  |  |  |  |  |  |  |  |  |

Table A.6: AST Data observed 3

| **Microbe** | **Isolate** | **IPM mean** | | **AMX mean** | | **N mean** | | **MRP mean** | | **CIP mean** | |
| --- | --- | --- | --- | --- | --- | --- | --- | --- | --- | --- | --- |
| Escherichia spp. | S1C1 | 29.0 | S | 30.3 | S | 15.0 | I | 35.0 | S | 16.3 | R |
| Shigella spp. | S1C2 | 29.7 | S | 0.0 | R | 30.0 | S | 32.7 | S | 37.7 | S |
| Escherichia spp. | S1C3 | 28.0 | S | 0.0 | R | 24.0 | S | 33.7 | S | 19.3 | R |
| Escherichia spp. | S1C4 | 31.0 | S | 0.0 | R | 16.7 | I | 36.3 | S | 12.3 | R |
| Shigella spp. | S1C5 | 25.7 | S | 0.0 | R | 25.3 | S | 31.0 | S | 29.3 | S |
| Escherichia spp. | S1C6 | 29.3 | S | 0.0 | R | 24.7 | S | 31.3 | S | 20.0 | R |
| Yersinia spp. | S1C7 | 24.3 | S | 14.0 | I | 25.7 | S | 24.7 | S | 29.3 | S |
| Yersinia spp. | S1C8 | 31.0 | S | 0.0 | R | 15.0 | I | 33.0 | S | 16.7 | R |
| Citrobacter spp. | S1I1 | 35.0 | S | 18.3 | S | 25.7 | S | 35.7 | S | 28.0 | S |
| Shigella spp. | S1I2 | 25.0 | S | 0.0 | R | 25.0 | S | 36.0 | S | 12.0 | R |
| Escherichia spp. | S2C1 | 32.7 | S | 0.0 | R | 22.0 | S | 34.0 | S | 10.7 | R |
| Escherichia spp. | S2C2 | 30.3 | S | 16.3 | I | 13.3 | R | 31.7 | S | 13.7 | R |
| Morganella spp. | S2C3 | 27.0 | S | 16.0 | I | 21.0 | S | 30.3 | S | 19.7 | R |
| Escherichia spp. | S2C4 | 25.3 | S | 10.0 | R | 13.3 | R | 29.0 | S | 14.0 | R |
| Escherichia spp. | S2C5 | 25.0 | S | 15.3 | I | 14.7 | I | 31.3 | S | 14.0 | R |
| Escherichia spp. | S2C6 | 25.7 | S | 15.7 | I | 13.3 | R | 32.0 | S | 13.3 | R |
| Escherichia spp. | S2C7 | 26.3 | S | 15.0 | I | 14.0 | R | 32.0 | S | 13.3 | R |
| Escherichia spp. | S2i2 | 27.7 | S | 0.0 | R | 13.7 | R | 32.7 | S | 17.3 | R |
| Escherichia spp. | S2i3 | 25.3 | S | 0.0 | R | 13.7 | R | 32.0 | S | 17.7 | R |
| Other | S3C1 |  |  | 11.3 | R | 20.3 | S | 32.3 | S | 25.0 | I |
| Escherichia spp. | S3C2 | 27.0 | S | 0.0 | R | 22.0 | S | 34.0 | S | 15.3 | R |
| Serratia spp. | S3C3 | 31.7 | S | 11.7 | R | 20.3 | S | 30.0 | S | 25.0 | I |
| Escherichia spp. | S3C4 |  |  | 0.0 | R | 20.0 | S | 31.0 | S | 9.3 | R |
| Unidentified | S3C5 | 32.7 | S | 0.0 | R | 20.7 | S | 28.7 | S | 23.3 | I |
| Unidentified | S3C6 | 31.0 | S | 15.7 | I | 21.3 | S | 32.7 | S | 22.0 | I |
| Other | S3C7 |  |  | 17.7 | S | 21.0 | S | 35.3 | S | 28.7 | S |
| Salmonella spp. | S3C8 |  |  | 5.7 | R | 20.7 | S | 30.0 | S | 20.7 | R |
| Escherichia spp. | S3C9 |  |  | 0.0 | R | 20.5 | S | 33.0 | S | 26.0 | S |
| Shigella spp. | S3C10 |  |  | 2.3 | R | 19.3 | S | 32.0 | S | 23.3 | I |
| Other | S3C11 | 31.0 | S | 16.3 | I | 25.7 | S | 37.3 | S | 27.3 | S |
| Salmonella spp. | S3C12 | 30.3 | S | 15.0 | I | 22.0 | S | 31.3 | S | 24.3 | I |
| Shigella spp. | S3C13 |  |  | 11.7 | R | 21.7 | S | 32.7 | S | 23.3 | I |
| Citrobacter spp. | S3C14 |  |  | 0.0 | R | 18.3 | S | 30.7 | S | 9.7 | R |
| Plesiomonas spp. | S3C16 |  |  | 3.0 | R | 19.3 | S | 35.7 | S | 17.0 | R |
| Escherichia spp. | S3I1 | 31.7 | S | 0.0 | R | 20.3 | S | 30.7 | S | 21.7 | I |
| Shigella spp. | S3I2 | 25.7 | S | 0.0 | R | 21.0 | S | 29.0 | S | 25.0 | I |
| Escherichia spp. | S3I3 | 30.0 | S | 0.0 | R | 19.7 | S | 30.7 | S | 10.0 | R |
| Shigella spp. | S3I4 | 30.7 | S | 22.3 | S | 22.3 | S | 35.7 | S | 30.0 | S |
| Escherichia spp. | S3I5 | 29.7 | S | 18.3 | S | 19.3 | S | 31.3 | S | 34.7 | S |
| Citrobacter spp. | S3I6 | 35.7 | S | 18.3 | S | 22.3 | S | 34.7 | S | 32.7 | S |
| Yersinia spp. | S3I7 | 31.3 | S | 19.0 | S | 20.0 | S | 30.7 | S | 26.0 | S |
| Shigella spp. | S4C1 | 36.7 | S | 0.0 | R | 21.7 | S | 36.3 | S | 17.0 | R |
| Yersinia spp. | S4C2 | 31.7 | S | 0.0 | R | 15.3 | I | 36.0 | S | 0.0 | R |
| Shigella spp. | S4C3 | 38.0 | S | 0.0 | R | 22.3 | S | 39.7 | S | 18.0 | R |
| Shigella spp. | S4C4 | 35.0 | S | 0.0 | R | 19.0 | S | 37.0 | S | 13.7 | R |
| Yersinia spp. | S4C5 | 36.3 | S | 4.7 | R | 19.3 | S | 36.0 | S | 14.7 | R |
| Shigella spp. | S4I1 | 35.0 | S | 0.0 | R | 19.3 | S | 35.0 | S | 14.0 | R |
| Escherichia spp. | S4I2 | 32.3 | S | 0.0 | R | 16.0 | I | 36.0 | S | 27.3 | S |
| Escherichia spp. | S4I3 | 32.0 | S | 0.0 | R | 15.0 | I | 32.7 | S | 30.0 | S |
| Escherichia spp. | S4I4 | 30.5 | S | 0.0 | R | 19.3 | S | 31.3 | S | 12.3 | R |
| Yersinia spp. | S4I5 | 29.5 | S | 6.5 | R | 18.5 | S | 31.5 | S | 15.5 | R |
| Escherichia spp. | S5C1 | 28.0 | S | 0.0 | R | 13.7 | R | 29.3 | S | 24.3 | I |
| Shigella spp. | S5C2 | 26.3 | S | 15.0 | I | 18.7 | S | 29.0 | S | 24.7 | I |
| Escherichia spp. | S5C3 | 33.3 | S | 0.0 | R | 15.7 | I | 35.0 | S | 7.3 | R |
| Escherichia spp. | S5C4 | 28.7 | S | 0.0 | R | 19.7 | S | 28.7 | S | 23.3 | I |
| Proteus spp. | S5C5 | 28.0 | S | 15.7 | I | 19.7 | S | 32.0 | S | 27.0 | S |
| Escherichia spp. | S5I1 | 28.0 | S | 0.0 | R | 18.7 | S | 28.0 | S | 20.0 | R |
| Escherichia spp. | S5I2 | 29.7 | S | 16.3 | I | 20.0 | S | 31.3 | S | 34.3 | S |
| Proteus spp. | S5I3 | 26.0 | S | 17.7 | S | 20.7 | S | 31.0 | S | 25.7 | I |
| Yersinia spp. | S5I4 | 27.3 | S | 0.0 | R | 20.0 | S | 29.7 | S | 24.7 | I |
| Proteus spp. | S5I5 | 31.3 | S | 9.0 | R | 22.0 | S | 32.3 | S | 30.0 | S |
| Escherichia spp. | S6C1 | 27.0 | S | 0.0 | R | 21.7 | S | 35.3 | S | 14.0 | R |
| Unidentified | S6C2 | 21.3 | I | 9.3 | R | 19.7 | S | 27.0 | S | 23.0 | I |
| Escherichia spp. | S6C3 | 23.7 | S | 0.0 | R | 15.0 | I | 33.0 | S | 14.0 | R |
| Citrobacter spp. | S6C4 | 28.7 | S | 18.3 | S | 24.0 | S | 35.0 | S | 25.0 | I |
| Unidentified | S6C5 | 21.7 | I | 0.0 | R | 21.3 | S | 27.3 | S | 21.0 | R |
| Escherichia spp. | S6I1 | 34.3 | S | 12.0 | R | 14.0 | R | 36.3 | S | 13.0 | R |
| Salmonella spp. | S6I2 | 28.3 | S | 19.7 | S | 25.7 | S | 37.7 | S | 30.7 | S |
| Citrobacter spp. | S6I3 | 29.3 | S | 21.0 | S | 25.3 | S | 36.3 | S | 35.3 | S |
| Escherichia spp. | S6I4 | 28.7 | S | 0.0 | R | 11.0 | R | 33.0 | S | 9.7 | R |
| Yersinia spp. | S6I5 | 28.0 | S | 18.0 | S | 21.3 | S | 33.7 | S | 9.3 | R |
| Enterobacter spp. | S7C1 | 25.7 | S | 0.0 | R | 19.0 | S | 28.0 | S | 28.7 | S |
| Klebsiella spp. | S7C2 | 27.3 | S | 8.7 | R | 21.0 | S | 30.3 | S | 31.7 | S |
| Proteus spp. | S7C3 | 26.7 | S | 2.3 | R | 23.3 | S | 30.7 | S | 25.0 | I |
| Escherichia spp. | S7C4 | 32.3 | S | 9.0 | R | 14.7 | I | 31.3 | S | 14.7 | R |
| Shigella spp. | S7C5 | 32.3 | S | 0.0 | R | 18.7 | S | 30.0 | S | 15.3 | R |
| Proteus spp. | S7I1 | 22.3 | I | 0.0 | R | 21.0 | S | 24.0 | S | 22.0 | I |
| Klebsiella spp. | S7I2 | 29.7 | S | 5.3 | R | 23.0 | S | 29.7 | S | 30.7 | S |
| Citrobacter spp. | S7I3 | 24.0 | S | 13.7 | I | 20.3 | S | 28.3 | S | 28.3 | S |
| Plesiomonas spp. | S7I4 | 33.7 | S | 0.0 | R | 24.0 | S | 23.0 | S | 25.0 | I |
| Escherichia spp. | S7I5 | 30.7 | S | 0.0 | R | 14.0 | R | 29.0 | S | 19.7 | R |
| Other | S8C1 | 29.0 | S | 0.0 | R | 16.0 | I | 29.7 | S | 16.7 | R |
| Escherichia spp. | S8C2 | 30.3 | S | 0.0 | R | 15.7 | I | 33.0 | S | 12.7 | R |
| Escherichia spp. | S8C3 | 29.3 | S | 0.0 | R | 23.0 | S | 34.7 | S | 23.7 | I |
| Shigella spp. | S8C4 | 29.7 | S | 22.7 | S | 20.7 | S | 35.7 | S | 27.0 | S |
| Yersinia spp. | S8C5 | 28.0 | S | 0.0 | R | 14.0 | R | 26.0 | S | 9.7 | R |
| Serratia spp. | S8I1 | 26.0 | S | 10.3 | R | 20.7 | S | 27.0 | S | 23.0 | I |
| Klebsiella spp. | S8I2 | 27.0 | S | 0.0 | R | 21.0 | S | 30.3 | S | 31.0 | S |
| Escherichia spp. | S8I3 | 29.7 | S | 0.0 | R | 16.3 | I | 19.7 | I | 24.3 | I |
| Proteus spp. | S8I4 | 44.3 | S | 25.7 | S | 27.3 | S | 39.0 | S | 39.3 | S |
| Plesiomonas spp. | S8I5 | 50.7 | S | 17.7 | S | 21.0 | S | 33.3 | S | 33.3 | S |
| Klebsiella spp. | S9C1 | 28.0 | S | 19.3 | S | 18.0 | S | 30.7 | S | 24.7 | I |
| Plesiomonas spp. | S9C2 | 29.7 | S | 0.0 | R | 21.7 | S | 31.0 | S | 31.3 | S |
| Pantoea spp. | S9C3 | 39.0 | S | 0.0 | R | 24.3 | S | 36.7 | S | 36.3 | S |
| Proteus spp. | S9C4 | 32.3 | S | 10.7 | R | 21.3 | S | 30.3 | S | 26.3 | S |
| Enterobacter spp. | S9C5 | 27.3 | S | 2.7 | R | 22.0 | S | 28.7 | S | 26.3 | S |
| Citrobacter spp. | S9I1 | 27.3 | S | 22.0 | S | 34.7 | S | 44.7 | S | 40.0 | S |
| Yersinia spp. | S9I2 | 34.3 | S | 3.3 | R | 25.0 | S | 36.7 | S | 30.0 | S |
| Citrobacter spp. | S9I3 | 25.3 | S | 10.7 | R | 23.3 | S | 29.0 | S | 26.7 | S |
| Proteus spp. | S9I4 | 48.7 | S | 24.0 | S | 22.5 | S | 43.0 | S | 34.0 | S |
| Klebsiella spp. | S9I5 | 32.0 | S | 0.0 | R | 23.7 | S | 34.3 | S | 28.7 | S |
| Other | S10C1 | 26.0 | S | 0.0 | R | 22.0 | S | 26.3 | S | 25.3 | I |
| Yersinia spp. | S10C2 | 31.3 | S | 0.0 | R | 24.3 | S | 32.3 | S | 17.7 | R |
| Citrobacter spp. | S10C3 | 30.7 | S | 14.3 | I | 16.7 | I | 32.7 | S | 24.3 | I |
| Escherichia spp. | S10C4 | 27.7 | S | 0.0 | R | 20.7 | S | 30.3 | S | 25.7 | I |
| Escherichia spp. | S10C5 | 26.0 | S | 0.0 | R | 21.0 | S | 28.3 | S | 24.3 | I |
| Proteus spp. | S10I1 | 29.0 | S | 0.0 | R | 13.7 | R | 33.7 | S | 25.7 | I |
| Citrobacter spp. | S11C1 | 28.3 | S | 4.3 | R | 22.7 | S | 30.3 | S | 28.3 | S |
| Proteus spp. | S11C2 | 25.7 | S | 0.0 | R | 17.0 | S | 24.3 | S | 23.3 | I |
| Proteus spp. | S11C3 | 26.7 | S | 7.0 | R | 17.7 | S | 31.3 | S | 21.3 | I |
| Proteus spp. | S11C4 | 25.0 | S | 0.0 | R | 23.0 | S | 31.7 | S | 28.7 | S |
| Enterobacter spp. | S11C5 | 29.3 | S | 0.0 | R | 21.0 | S | 28.7 | S | 24.3 | I |
| Pantoea spp. | S11I1 | 31.0 | S | 0.0 | R | 22.0 | S | 28.7 | S | 25.0 | I |
| Shigella spp. | S11I2 | 23.7 | S | 0.0 | R | 22.3 | S | 28.7 | S | 25.0 | I |
| Escherichia spp. | S11I3 | 28.7 | S | 18.3 | S | 21.0 | S | 32.3 | S | 31.7 | S |
| Escherichia spp. | S11I4 | 27.3 | S | 2.7 | R | 19.3 | S | 29.3 | S | 10.3 | R |
| Salmonella spp. | S11I5 | 34.0 | S |  |  |  |  |  |  |  |  |
| Escherichia spp. | P1C1 | 30.3 | S | 0.0 | R | 20.0 | S | 30.7 | S | 26.3 | S |
| Escherichia spp. | P1C2 | 31.0 | S | 0.0 | R | 20.7 | S | 31.0 | S | 20.3 | R |
| Citrobacter spp. | P1C3 | 31.0 | S | 0.0 | R | 22.0 | S | 33.3 | S | 20.3 | R |
| Klebsiella spp. | P1C4 | 29.0 | S | 3.3 | R | 20.0 | S | 30.0 | S | 30.3 | S |
| Plesiomonas spp. | P1C5 | 30.0 | S | 0.0 | R | 13.7 | R | 30.7 | S | 25.0 | I |
| Shigella spp. | P1I1 | 31.3 | S | 4.0 | R | 20.3 | S | 32.7 | S | 27.7 | S |
| Unidentified | P1I2 | 28.3 | S | 0.0 | R | 20.3 | S | 31.3 | S | 26.0 | S |
| Unidentified | P1I3 | 27.7 | S | 0.0 | R | 19.0 | S | 29.0 | S | 27.7 | S |
| Escherichia spp. | P1I4 | 31.3 | S | 0.0 | R | 20.7 | S | 31.7 | S | 29.7 | S |
| Klebsiella spp. | P1I5 | 37.7 | S | 14.3 | I | 24.7 | S | 32.7 | S | 27.0 | S |
| Salmonella spp. | P2C1 | 21.3 | I | 0.0 | R | 19.0 | S | 26.0 | S | 26.3 | S |
| Plesiomonas spp. | P2C2 | 23.0 | S | 2.7 | R | 18.7 | S | 31.3 | S | 20.0 | R |
| Citrobacter spp. | P2C3 | 29.3 | S | 18.0 | S | 20.3 | S | 34.7 | S | 36.3 | S |
| Escherichia spp. | P2C4 | 28.7 | S | 0.0 | R | 13.0 | R | 32.7 | S | 2.7 | R |
| Escherichia spp. | P2I1 | 28.3 | S | 18.0 | S | 20.7 | S | 30.3 | S | 32.0 | S |
| Escherichia spp. | P2I2 | 26.0 | S | 3.7 | R | 21.0 | S | 31.0 | S | 27.3 | S |
| Escherichia spp. | P2I3 | 17.3 | R | 16.7 | I | 20.3 | S | 33.0 | S | 34.0 | S |
| Plesiomonas spp. | P2I4 | 21.3 | I | 0.0 | R | 21.0 | S | 26.0 | S | 22.3 | I |
| Yersinia spp. | P2I5 | 18.7 | R | 0.0 | R | 19.3 | S | 23.3 | S | 19.7 | R |
| IPM=Imipenem, AMX= Amoxicillin, N=Neomycin, MRP=Meropenem, CIP=Ciprofloxacin | | | | | | | | | | | |
|  |  |  |  |  |  |  |  |  |  |  |  |
